# Supplementary material for: Toxic PARP trapping upon cAMP-induced DNA damage reinstates the efficacy of endocrine therapy and CDK4/6 inhibitors in treatment-refractory ER+ breast cancer
Source: Nat Commun. 2023 Nov 2;14:6997. doi: 10.1038/s41467-023-42736-y (PMC10620179; doi:10.1038/s41467-023-42736-y)
Supplement: Supplementary file 1 — Supplementary Information [file 41467_2023_42736_MOESM1_ESM.docx]

**SUPPLEMENTARY INFORMATION**

**Toxic PARP trapping upon cAMP-induced DNA damage reinstates the efficacy of endocrine therapy and CDK4/6 inhibitors in treatment-refractory ER+ breast cancer**

Ozge Saatci^1,2^, Metin Cetin^1,2^, Meral Uner^3^, Unal Metin Tokat^4^, Ioulia Chatzistamou^5^, Pelin Gulizar Ersan^2^, Elodie Montaudon^6^, Aytekin Akyol^3^, Sercan Aksoy^7^, Aysegul Uner^3^, Elisabetta Marangoni^6^, Mathew Sajish^2^, Ozgur Sahin^1,2*^

^1^Department of Biochemistry and Molecular Biology, Hollings Cancer Center, Medical University of South Carolina, Charleston, SC, 29425, USA

^2^Department of Drug Discovery and Biomedical Sciences, University of South Carolina, Columbia, SC, 29208, USA

^3^Department of Pathology, Faculty of Medicine, Hacettepe University, 06100, Ankara, TURKEY

^4^Department of Molecular Biology and Genetics, Bilkent University, Ankara, 06800, TURKEY

^5^Department of Pathology, Microbiology & Immunology, University of South Carolina, Columbia, SC, 29208, USA

^6^Translational Research Department, Institut Curie, PSL Research University, Paris, 75005, FRANCE

^7^Department of Medical Oncology, Hacettepe University Cancer Institute, 06100, Ankara, TURKEY

**Keywords:** Endocrine therapy/CDK4/6 inhibitor/overcoming resistance/ DNA damage/BRCAness/PARP trapping/cAMP/ROS

**^*^Corresponding author**

Ozgur Sahin, PhD

Professor and SmartState Endowed Chair

Department of Biochemistry and Molecular Biology

Hollings Cancer Center

Medical University of South Carolina

86 Jonathan Lucas Street, Room HO712F,

Charleston, SC 29425

Phone: +1-843-792-0166

E-mail: [sahin@musc.edu](mailto:sahin@musc.edu)

**SUPPLEMENTARY METHODS**

**Transfection with siRNAs and overexpression vectors**

c-JUN-, PDE4D-, ESR1 and PARP1-specific siRNAs were purchased from Dharmacon (**Supplementary Table 3**). Transfections were done with 40 nmol/L (c-JUN, PDE4D and ESR1) or 20 nmol/L (PARP1) siRNA using Lipofectamine 2000 (Invitrogen) according to manufacturer's instructions. PDE4D cDNA (NM_006203.4) and *BRCA1* cDNA (NM_007300.4) present in pcDNA3.1+/C-(K) DYK mammalian expression vector (GenScript) was used for overexpression experiments. Briefly, 15 ng (for 96-well plate) or 500 ng (for 6-well plate) of PDE4D plasmid DNA or 500 ng (for 6-well plate) of *BRCA1* plasmid DNA were transiently transfected using Lipofectamine 2000 (Invitrogen) according to manufacturer's instructions.

**Inhibitor, hormone or growth factor treatments and cell proliferation assays**

For cAMP measurements, parental or SOC resistant MCF-7 or T47D cells were treated with 7.5 uM of tamoxifen, fulvestrant or 2.5 uM palbociclib for 2 hours. For cell viability assays, treatment with the individual or combination therapies were done at the same time for a total of 72 hours. The synergy between two combined drugs was assessed based on the Bliss Independence model^1^. The calculation CI was done using the following formula: CI = (EA+EB-EA*EB)/EAB. Here, EA and EB are the effects of reagents A and B on cell viability alone, while EAB is the inhibitory effect when A and B are combined. For the ER activity and cell growth assay under E2 stimulation, MCF-7 cells were first starved in charcoal-stripped FBS media for a week, followed by stimulation with 10 or 20 nM estradiol (Sigma) with or without different drugs at the same time. To test the effects of PDE4D on E2-induced ER phosphorylation, starved cells were pretreated with 1 uM tamoxifen, 20 ug/mL GebR-7b or 2 uM ulixertinib for 20 min followed by stimulation with 20 nM E2 for 5 min or 24 hours. For EGF stimulation experiments, cells were first starved in serum-free media for 24 hours, followed by stimulation with 20 nM EGF for 4 or 24 hours. The ROS scavenger, NAC was given at a dose of 10 mM, 1 hour before treatment with SOC. HBCx-118 and HBCx-131 organoid treatments were done for a total 7 days.

**Quantitative RT-PCR analysis**

Total RNA was extracted from cultured cells using TRIsure (Bioline), and cDNAs were generated using RevertAid RT Reverse Transcription Kit (Life Technologies). qRT-PCR analysis was performed with gene-specific primers using LightCycler 480 SYBR Green I Master kit (Roche). *HPRT1* and *ACTB* were used as housekeeping genes. The average Ct value was calculated from triplicates of each sample, and the relative mRNA expression was determined. Sequences of the qRT-PCR primers are listed in **Supplementary Table 3**.

**HR reporter assay**

The homologous recombination repair capacity of the models was assessed using Homologous Recombination Assay Kit (Norgen Biotek, catalog no: 35600) by following manufacturer’s instructions. Briefly, cells were seeded in 24-well plates and transfected with the dl1 and dl2 plasmids on the next day. 12 hours after transfection, cells were treated with 20 or 50 uM of etoposide for 24 hours and DNA was isolated. qRT-pCR was done using the Assay primers and Universal Primers provided with the kit to amplify the HR product and the total plasmids, respectively.

**Chromatin immunoprecipitation (ChIP) assay**

ChIP assay was done as described previously^2,3^. Briefly, MCF-7 cells that were grown to 70% confluency (for ER ChIP) or T47D cells grown to 50% confluency and then serum-starved for 24 hours, followed by 16 hours stimulation with 20 nM EGF (for c-Jun ChIP) were crosslinked with 1% formaldehyde for 10 min followed by quenching with 125 mM glycine for 5 min. Cells were lysed in 500 µL lysis buffer, and nuclear lysates were extracted. Following the sonication to shear DNA, the chromatin was incubated with the beads together with the ER antibody (Active Motif, 61035) or c-Jun antibody (Cell Signaling, 9165) at + 4 °C, overnight with slow agitation. Beads with no antibody was used as the negative control. Samples were washed with low and high salt wash buffers, and Proteinase K treatment was done for 2 h at 62 °C with shaking. Samples were incubated at 95 °C for 10 min and separated from the beads using a magnetic separator. DNA was isolated, and RT-PCR was performed using primers targeting the predicted ER or c-Jun binding sites on the PDE4D promoters that transcribe different isoforms (**Supplementary Table 3**; Promoter P1: PDE4D5; Promoter P2: PDE4D7; and Promoter P6: PDE4D4; Promoter P3: PDE4D3; Promoter P7: PDE4D8; and Promoter P8: PDE4D9). Primers for PGR and GREB1 were used as positive control in ER ChIP while primers for BCLXL and ERCC1 were used as positive control in c-Jun ChIP. The results were normalized to bead only control and represented as fold enrichment.

**Western blotting**

Western blotting was done as describe previously^3-5^. Briefly, total protein was extracted using RIPA buffer (150 mmol/L NaCl, 50 mmol/L Tris base pH 8.0, 1 mmol/L EDTA, 0.5% sodium deoxycholate, 1% NP40, 0.1% SDS, 1 mmol/L DTT, and 1 mmol/L Na3VO4) supplemented with Complete Protease Inhibitor (Roche). Protein concentrations were measured using BCA Protein Assay (Thermo Scientific). Equal amounts of protein lysates (15–20 μg) were separated on a 10% SDS-PAGE, transferred onto polyvinylidene difluoride membrane (Bio-Rad) and incubated with primary antibodies listed in **Supplementary Table 4**. The blots were developed using enhanced chemiluminescence (ECL) detection kit (Amersham Biosciences) after incubation with horseradish peroxidase–conjugated secondary antibody. β-Actin was used as a loading control.

**EGFR array**

The EGFR array was performed using the Human EGFR Phosphorylation Array C1 from RayBiotech according to the manufacturer's instructions.

**Intracellular cAMP measurements**

To measure intracellular cAMP levels, parental and SOC resistant MCF-7 or T47D cells that were treated with SOC were washed once with PBS and detached using Tryple. 3,000 MCF-7 and 15,000 T47D cells/well were collected in eppendorfs and lysed with ice-cold 2.5 mol/L perchloric acid followed by neutralization with 2 M KOH. Cells were incubated on ice for 30 minutes and then centrifuged at 10,000 rpm for 10 min to collect the clear lysate. Twenty microliters of the clear lysate were transferred per well of a white plate, and cAMP measurement was done using the LANCE Ultra cAMP detection kit (PerkinElmer) according to the manufacturer's protocol.

**Intracellular ROS measurements**

The intracellular ROS was measured using the DCFDA / H2DCFDA - Cellular ROS Assay Kit from Abcam according to the manufacturer's protocol. Briefly, 8,000 T47D cells were seeded on clear bottom, black wall plates and next day, stained with 20 µM DCFDA for 1 hour. Then, 7.5 µM tamoxifen or fulvestrant or 2.5 µM palbociclib were added on top, and fluorescence was detected by a Multimode SpectraMax plate reader at 37 ^0^C with excitation/emission at 485 nm/535 nm.

**Mitochondrial ROS measurements**

Mitochondrial ROS measurement was done using the MitoSOX™ Red Mitochondrial Superoxide Indicator. Briefly, 8,000 T47D cells were seeded on clear bottom, black wall plates and next day, stained with 5 µM MitoSOX for 15 min. Then, 7.5 µM tamoxifen or fulvestrant or 2.5 µM palbociclib were added on top, and fluorescence was detected by a Multimode SpectraMax plate reader at 37 °C with excitation/emission at 510 nm/580 nm for a total of 2 hours. For testing the effects of PKA inhibition, T47D cells were pretreated with 50 µM of the PKA inhibitor, Rp-Cyclic AMPS for 1 hour. For testing the effects of PDE4D overexpression, one day prior to seeding onto clear bottom, black wall plates, T47D cells were transfected with 500 ng of the PDE4D plasmid DNA in 6well format.

**Immunofluorescence staining**

Cells were seeded in 6-well plates on cover slips. Next day after seeding, cells were treated with SOC therapies or etoposide for 4 hours. Fixation was done with 4% PFA for 15 min at room temperature, followed by permeabilization with 0.25% Triton X-100 for 5 min at room temperature. Blocking was done with 3% BSA-PBS for 1 hour. Primary antibodies, γ-H2AX (1:500) and RAD51 (1:150) were diluted in 3% BSA-PBS and cells were incubated with the primary antibodies for 1.5 hours at room temperature. Secondary antibody solution was prepared in 3% BSA-PBS at a dilution of 1:1000 and incubation of cells with the secondary antibodies was done for 1 hour at room temperature. DAPI was used for nuclear counterstaining for 5 min at room temperature. Cover slips were mounted with ProLong™ Glass Antifade Mountant (ThermoScientific). Imaging was done using Zeiss LSM700 Confocal Microscopy. The number of cells with γ-H2AX foci and those that are also RAD51-positive were counted together with the total number of cells in 4 different regions with a minimum of 100 cells in each region using ImageJ software.

**Immunohistochemistry**

Tumor samples collected from HBCX-118 PDXs after treatments were fixed in 10% formalin and processed into paraffin blocks. Histologic sections were taken with 5-μm thickness and deparaffinized. All sections were stained for Haematoxylin & Eosin, Ki-67 (Abcam, ab16667) and TUNEL (In Situ Cell Death Detection Kit, Roche). For DCFDA staining, cryosections were prepared and staining was performed as previously reported^6^. For the staining of PDE4D protein in tumor samples, antigen retrieval was done for 10 min with the EDTA buffer, and slides were incubated with the PDE4D antibody (Proteintech, clone 12918) at a dilution of 1:100 for 20 minutes at room temperature using Leica BOND-MAX/ISH automated immune-stainer.

**
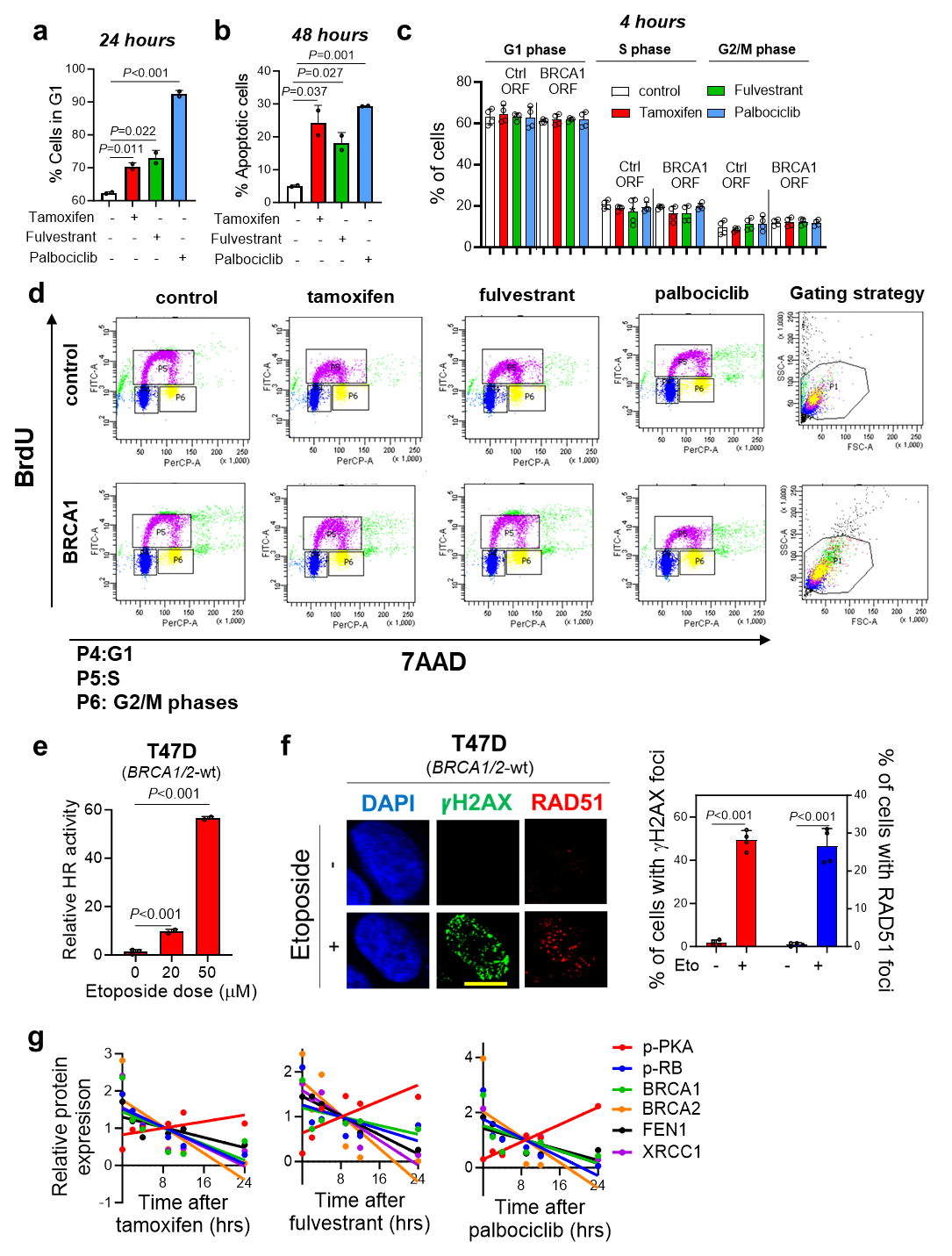
SUPPLEMENTARY FIGURES**

**Supplementary Figure 1. The effects of SOC therapy on cell cycle distribution (upon short-term and long-term treatment), apoptosis, PKA activation and DNA repair proteins, and determination of the HR proficiency of T47D cells. a, b** Cell cycle assay (a) and Annexin V/DAPI staining (b) in T47D cells treated with SOC for 24 and 48 hours, respectively (n=2). **c** Percentage of cells found in G1, S and G2/M phases of the cell cycle upon SOC treatment for 4 hours in the absence or presence of *BRCA1* ORF as determined by BrdU/7AAD staining (n=4). **d** Dot plots of cells stained with BrdU/7AAD after 4 hours treatment with SOC in the absence or presence of *BRCA1* ORF. Gating strategy is provided on the right. **e** Relative homologous recombination (HR) activity in T47D (*BRCA1/2* wt) cells upon treatment with increasing doses of etoposide for 12 hours (n=2, 3). **f** IF staining of γ-H2AX (S139) (green) and RAD51 (red) in T47D (*BRCA1/2* wt) cells upon treatment with etoposide for 4 hours. The quantification of γ-H2AX positive cells and those that are RAD51 positive are given at the right panel (n=4 different areas, with at least 100 cells per area). DAPI (blue) was used to stain the nucleus. **g** Quantification of p-PKA, p-RB, BRCA1, BRCA2 FEN1 and XRCC1 band intensities relative to actin in SOC-treated T47D cells in a time-dependent manner from Figure 1f. Data are presented as mean values ± standard deviation (SD). *P*-values were calculated with the unpaired, two-tailed Student’s t test. µM: micromolar, for all figures. Source data for this figure are provided as a Source Data file.


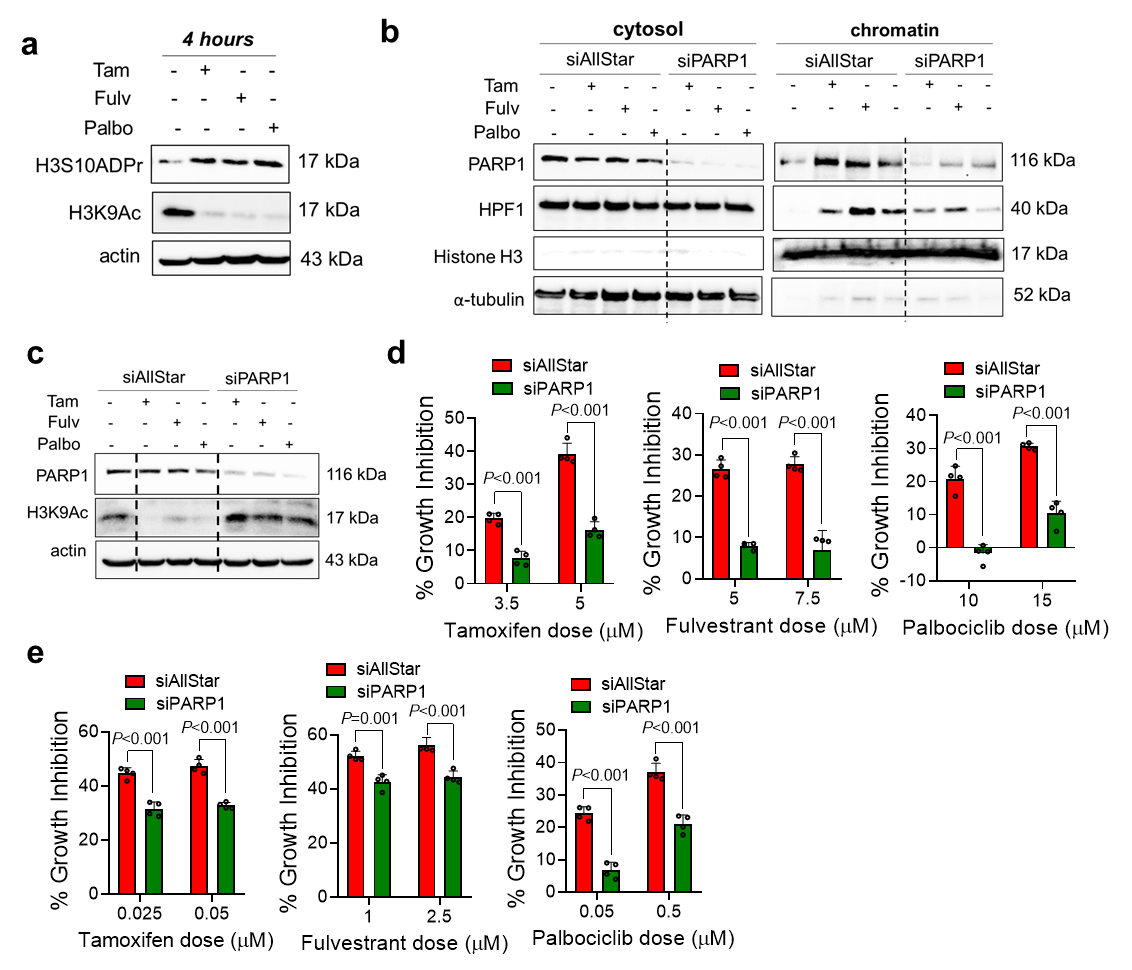


**Supplementary Figure 2. SOC induces H3S10 ADPR and reduces H3K9Ac upon toxic PARP1 trapping while PARP1 knockdown reduces PARP1 trapping, restores H3K9Ac and alleviates cell killing by SOC treatment.** **a** Western blot analysis of H3S10 ADPR and H3K9Ac in T47D cells treated with SOC for 4 hours. **b** Chromatin fractionation in T47D cells transfected with siRNA against PARP1 and treated with SOC for 2 hours. Histone H3 was used as the loading control for chromatin fraction, and α-tubulin was used as the loading control for cytosol fraction. **c** Western blot analysis of H3K9Ac upon 4 hours of SOC treatment in T47D cells transfected with siPARP1. **d, e.** Percentage growth inhibition in MCF7 (d) and T47D (e) cells transfected with siPARP1 and treated with SOC for 72 hours (n=4). Data are presented as mean values ± standard deviation (SD). *P*-values were calculated with the unpaired, two-tailed Student’s t test. Experiments in a-c are repeated twice with similar results. Source data for this figure are provided as a Source Data file.


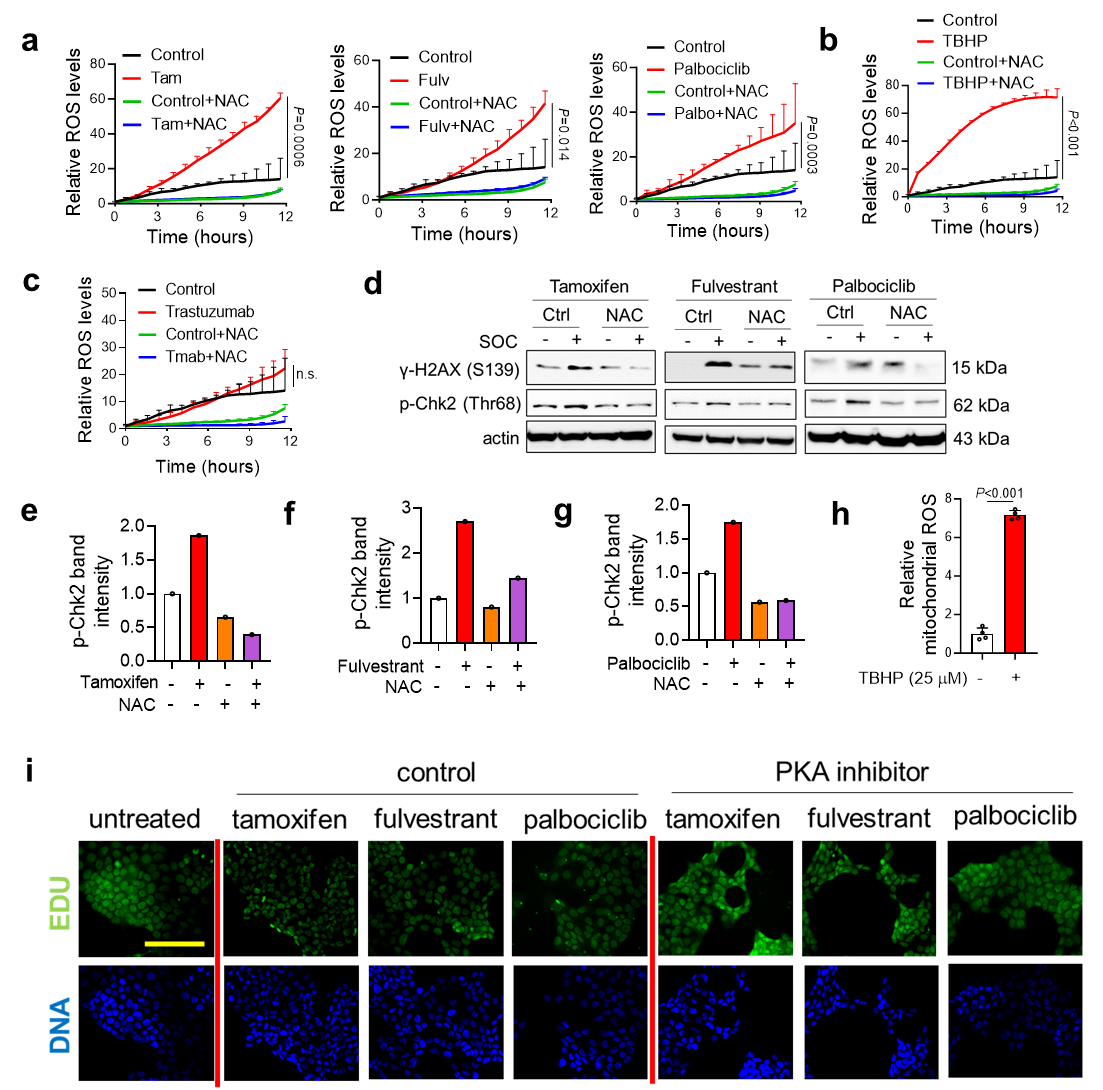


**Supplementary Figure 3. SOC therapy induces DNA damage and G1 arrest in a ROS and PKA-dependent manner. a-c** Time-dependent ROS generation in T47D cells upon treatment with SOC (a) or 25 uM TBHP (positive control) (b) or 1 ug/m trastuzumab (negative control) (c) (n=3). The control groups for a-c are the same as these experiments were done at the same time. **d** Western blot analysis of γ-H2AX (S139) and p-Chk2 (Thr68) in SOC-treated T47D cells with or without 10 mM of NAC. NAC (N-acetylcystein): ROS scavenger. Actin is used as the loading control. Experiment is repeated twice with similar results. **e-g** Quantification of the p-Chk2 (Thr68) band intensities relative to the housekeeping protein, actin upon tamoxifen (e), fulvestrant (f) and palbociclib (g) treatment in T47D cells with or without 10 mM of NAC. **h** MitoSOX staining in T47D cells treated with 25 uM of TBHP (positive control). **i** EdU staining (green) of T47D cells upon SOC treatment with or without the PKA inhibitor. DAPI (blue) shows the nucleus. Scale bar = 200 µm. Data are presented as mean values ± standard deviation (SD). *P*-values were calculated with paired, two-tailed Student’s t test. n.s., not significant (*P*>0.05). Source data for this figure are provided as a Source Data file.

**
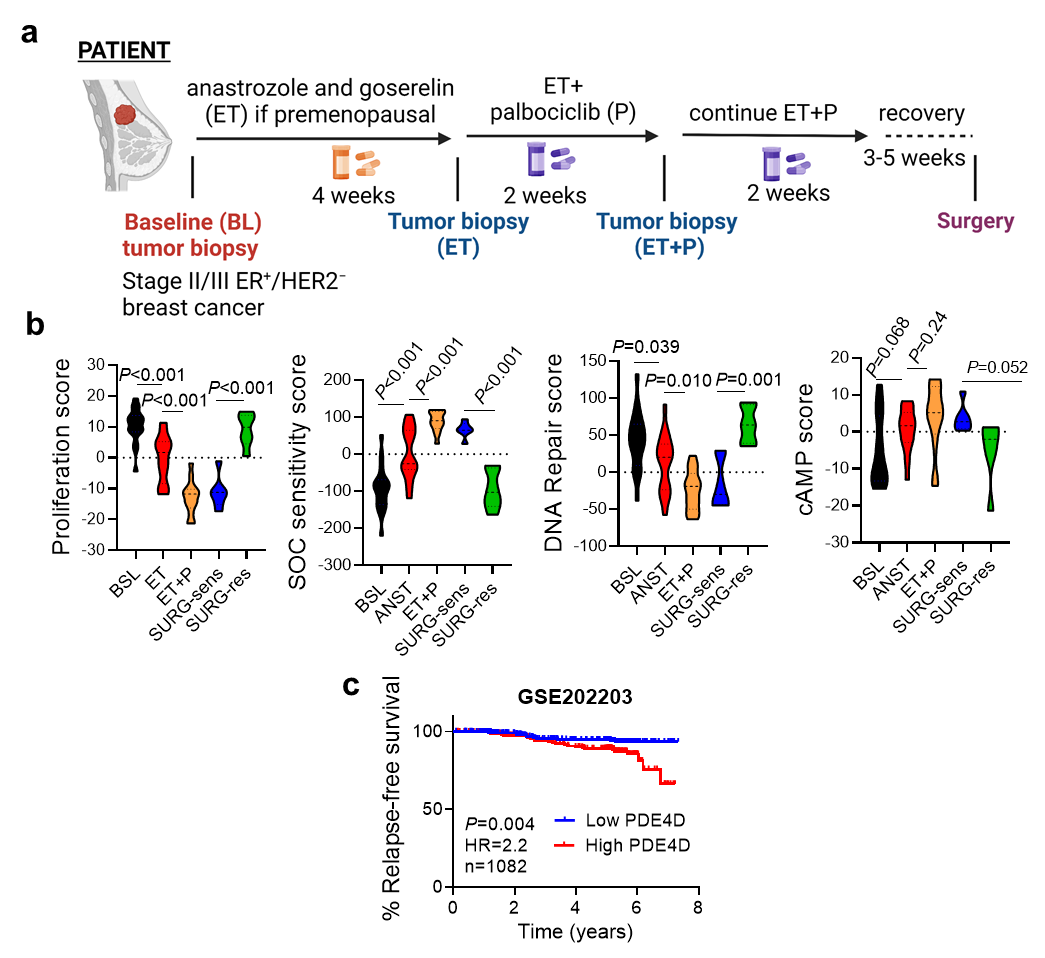
**

**Supplementary Figure 4. The dynamic changes of proliferation, DNA repair, SOC sensitivity and cAMP scores over the course of treatment and the association of PDE4D expression with clinical outcome of ER+ breast cancer patients. a** Scheme of study design and treatment from GSE93204. During the recovery phase, patients continued to receive ET, while only those recovered in terms of blood cell count received one last cycle of palbociclib 2 weeks before surgery. ET: endocrine therapy. P: palbociclib. This figure is created with Biorender.com. **b** Levels of proliferation score, SOC sensitivity score, DNA repair score and cAMP score in ER+ breast cancer patients from GSE93204 who were treated with endocrine therapy (ET), followed by addition of palbociclib (P) upon endocrine resistance development and finally underwent surgery. SENS represents sensitive patients at surgery while RES represents resistant patients. **c** Kaplan Meier relapse-free survival analysis in primary endocrine-treated ER+ breast cancer patients from GSE202203 based on PDE4D mRNA expression. Significance for the Kaplan Meier survival graphs was calculated with Log-rank test. *P*-values were calculated with the unpaired, two-tailed Student’s t test. * *P*<0.05, ***P*<0.01. Source data for this figure are provided as a Source Data file.

**
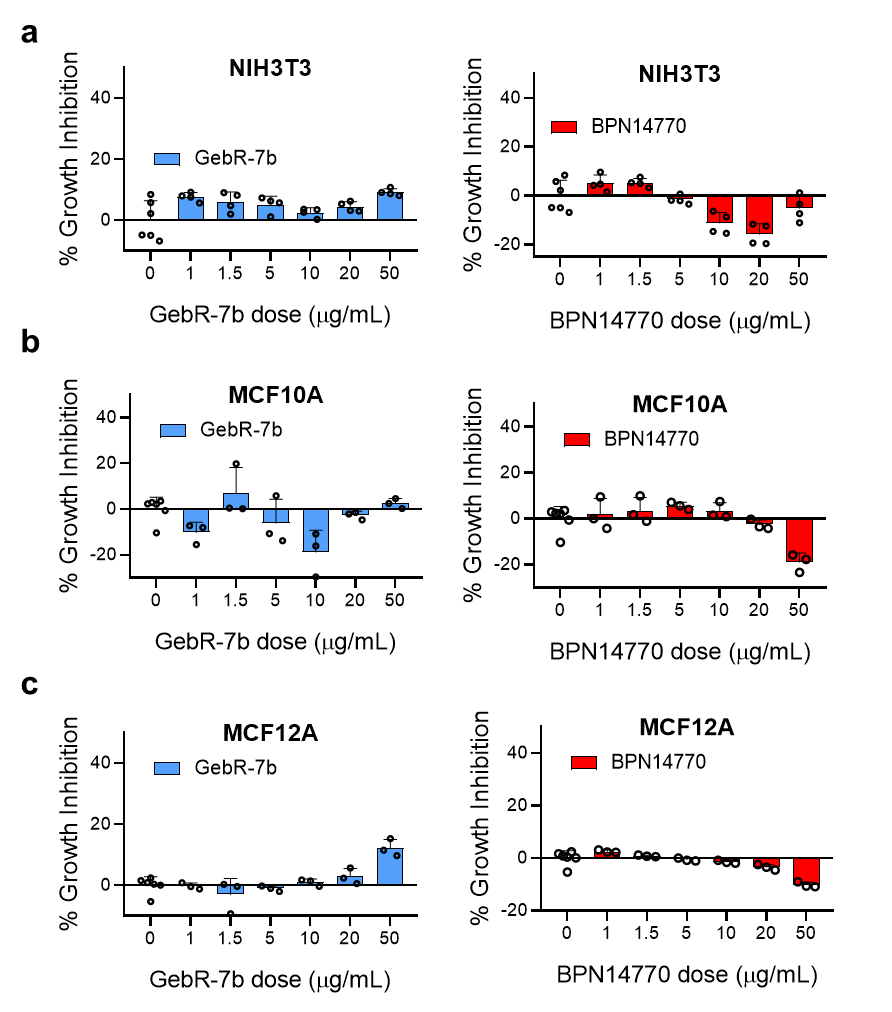
**

**Supplementary Figure 5. Testing potential cytotoxicity of PDE4D inhibition in normal mouse and human cells. a-c** Percentage growth inhibition in normal mouse NIH3T3 (a) and normal human ER- MCF10A (b) and MCF12A (c) cells treated with PDE4D inhibitor, GebR-7b (left panel) and BPN14770 (right panel) (n=3-6). Data are presented as mean values ± standard deviation (SD). Source data for this figure are provided as a Source Data file.

**
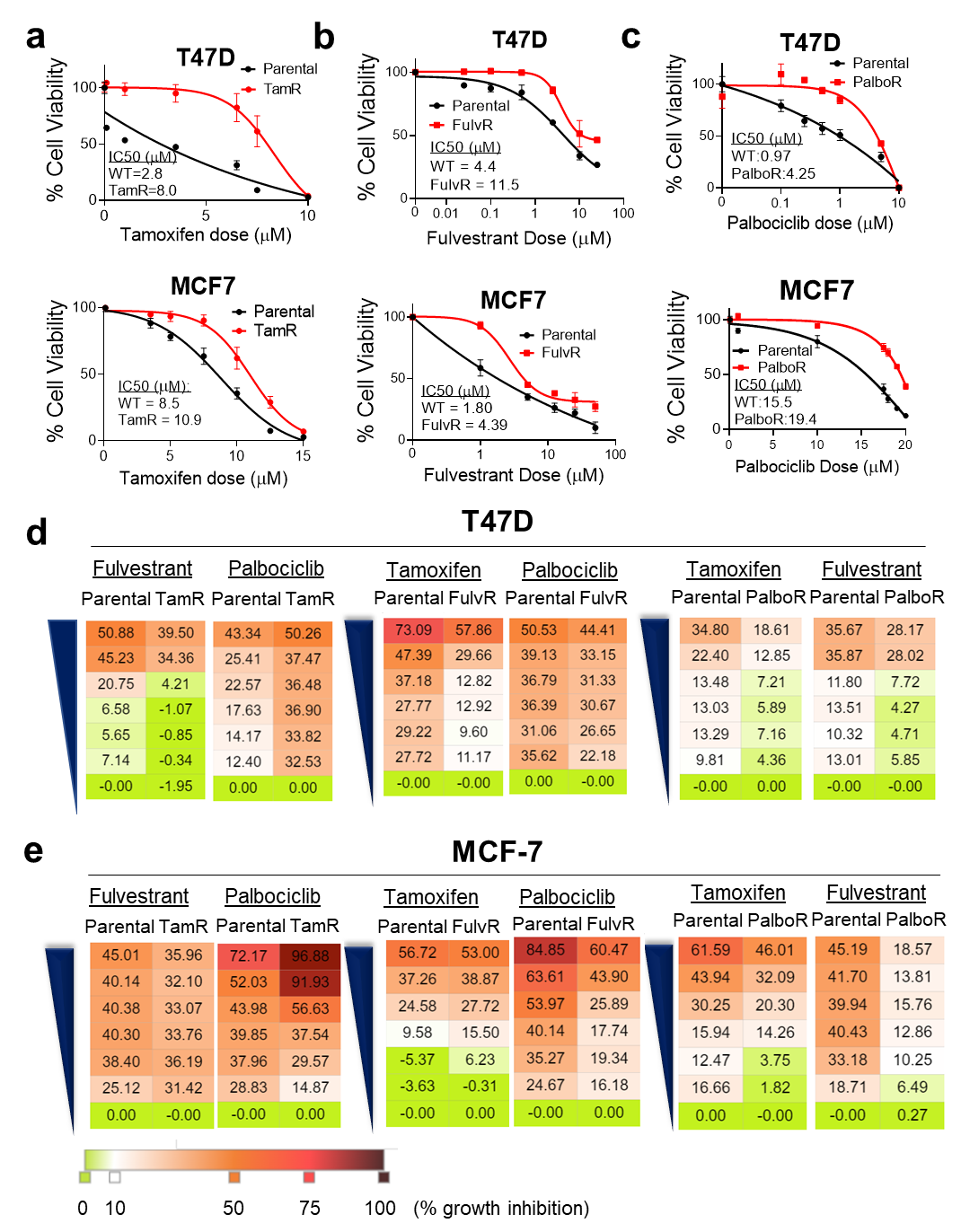
**

**Supplementary Figure 6. Dose response curves of acquired SOC resistant ER+ cell line models and their cross-resistance status.** **a-c** Percent cell viability in parental vs. resistant T47D and MCF-7 cells treated with increasing doses of tamoxifen (a), fulvestrant (b) or palbociclib (c) for 72 hours (n=4, 6). **d, e** Percent growth inhibition in parental and resistant T47D (D) and MCF-7 (E) cells treated with all SOC therapy at increasing doses to assess cross resistance (tamoxifen: 0.01-12.5 µM; fulvestrant: 0.05-50 µM; palbociclib: 0.05-20 µM) (n=4, 6). The numbers indicate % growth inhibition which are colored based on the scale bar provided below the figure. Data are presented as mean values ± standard deviation (SD). Source data for this figure are provided as a Source Data file.

**
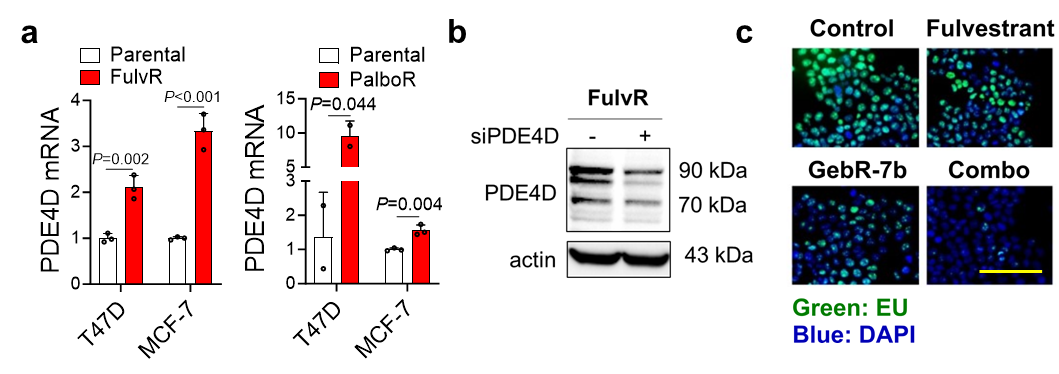
**

**Supplementary Figure 7. PDE4D is upregulated in SOC resistant cell line and PDX models, and its inhibition blocks transcription.** **a** qRT-PCR analysis of PDE4D in SOC parental vs. resistant cells (n=2-3). **b** Western blot analysis of PDE4D in siPDE4D-transfected T47D FulvR cells. Actin is used as the loading control. **c** Representative images of the EU staining (green) in T47D FulvR cells treated with fulvestrant and GebR-7b, alone or in combination. DAPI (blue) shows the nucleus. Scale bar=100 µm. Data are presented as mean values ± standard deviation (SD). *P*-values were calculated with the unpaired, two-tailed Student’s t test. Source data for this figure are provided as a Source Data file.


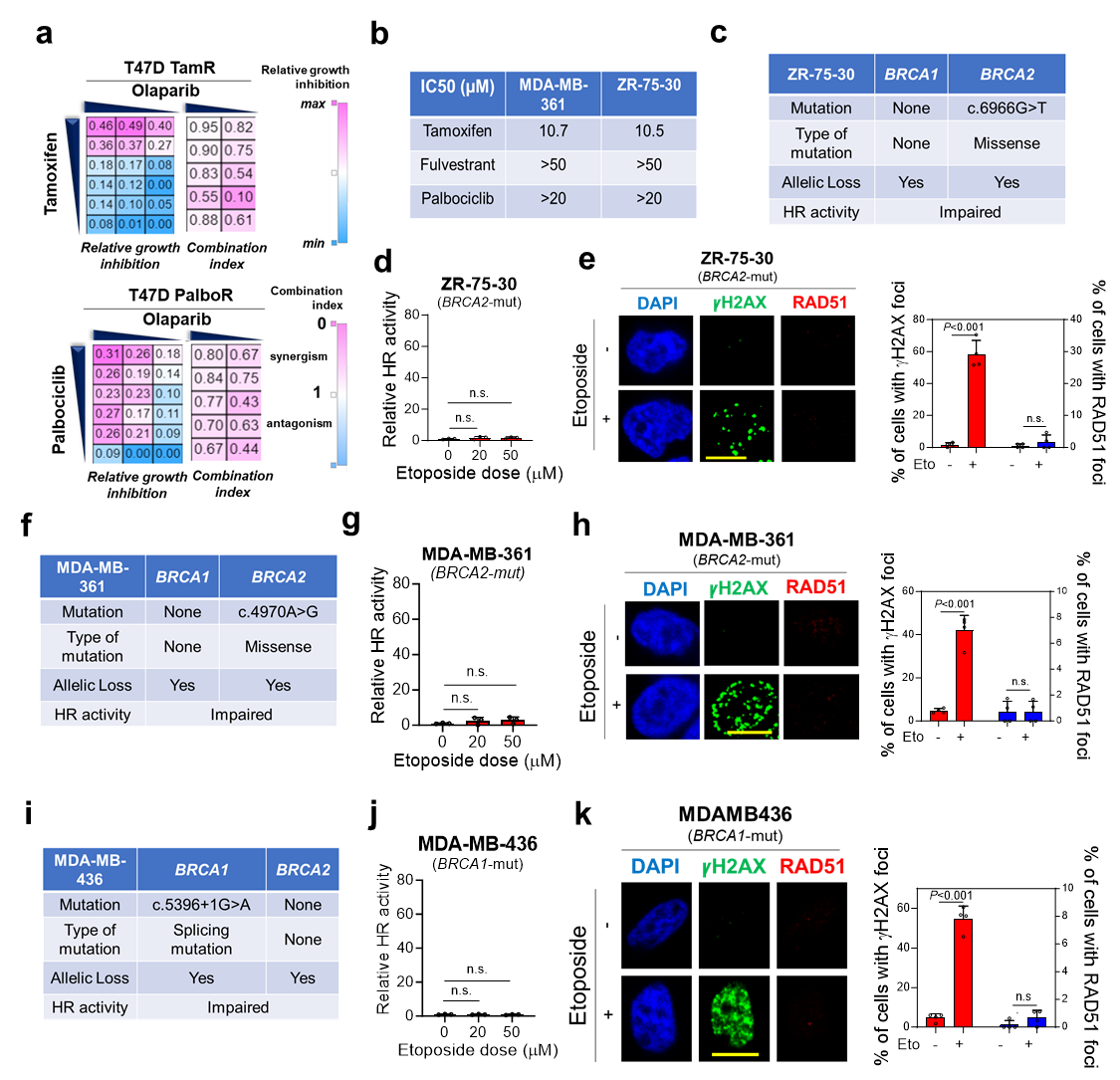


**Supplementary Figure 8. Testing the PARP1 inhibition-mediated SOC sensitization and the characterization of the *BRCA1/2*-mutant SOC resistant cells in terms of SOC response and homologous recombination (HR) proficiency. a** Heatmaps of relative growth inhibition and combination indices in SOC resistant cells treated with the combination of different SOC therapies (tamoxifen: 2, 3, 4, 5, 6 µM; palbociclib: 0.25, 0.5, 1, 2.5, 3.5 µM) with olaparib (5, 7.5 µM). The scale bars for the growth inhibition and combination index matrices are provided at the right-hand side, here and for all heatmaps. **b** Table of IC50 values of SOC therapies in *BRCA*-mut MDA-MB-361 and ZR-75-30 cells. **c, f, i** Tables of the *BRCA1 and BRCA2* mutations, mutation types, the allelic changes, and the HR activity in ZR-75-30 (c), MDA-MB-361 (f) and MDA-MB-436 (i) cell lines. **d, g, j** Relative homologous recombination (HR) activity in ZR-75-30 *(BRCA2*-mut) (d), MDA-MB-361 (*BRCA2*-mut) (g), and MDA-MB-436 (*BRCA1*-mut) (j) cells upon treatment with increasing doses of etoposide for 12 hours (n=3). **e, h, k** IF staining of γ-H2AX (S139) (green) and RAD51 (red) in ZR-75-30 *(BRCA2*-mut) (e), MDA-MB-361 (*BRCA2*-mut) (h) and MDA-MB-436 (*BRCA1*-mut) (k) cells upon treatment with etoposide for 4 hours. The quantification of γ-H2AX positive cells and those that are RAD51 positive are given at the right panel (n=4 different areas, with at least 100 cells per area). DAPI (blue) was used to stain the nucleus. Data are presented as mean values ± standard deviation (SD). *P*-values were calculated with the unpaired, two-tailed or one-tailed Student’s t test. n.s., not significant (*P*>0.05). Source data for this figure are provided as a Source Data file.


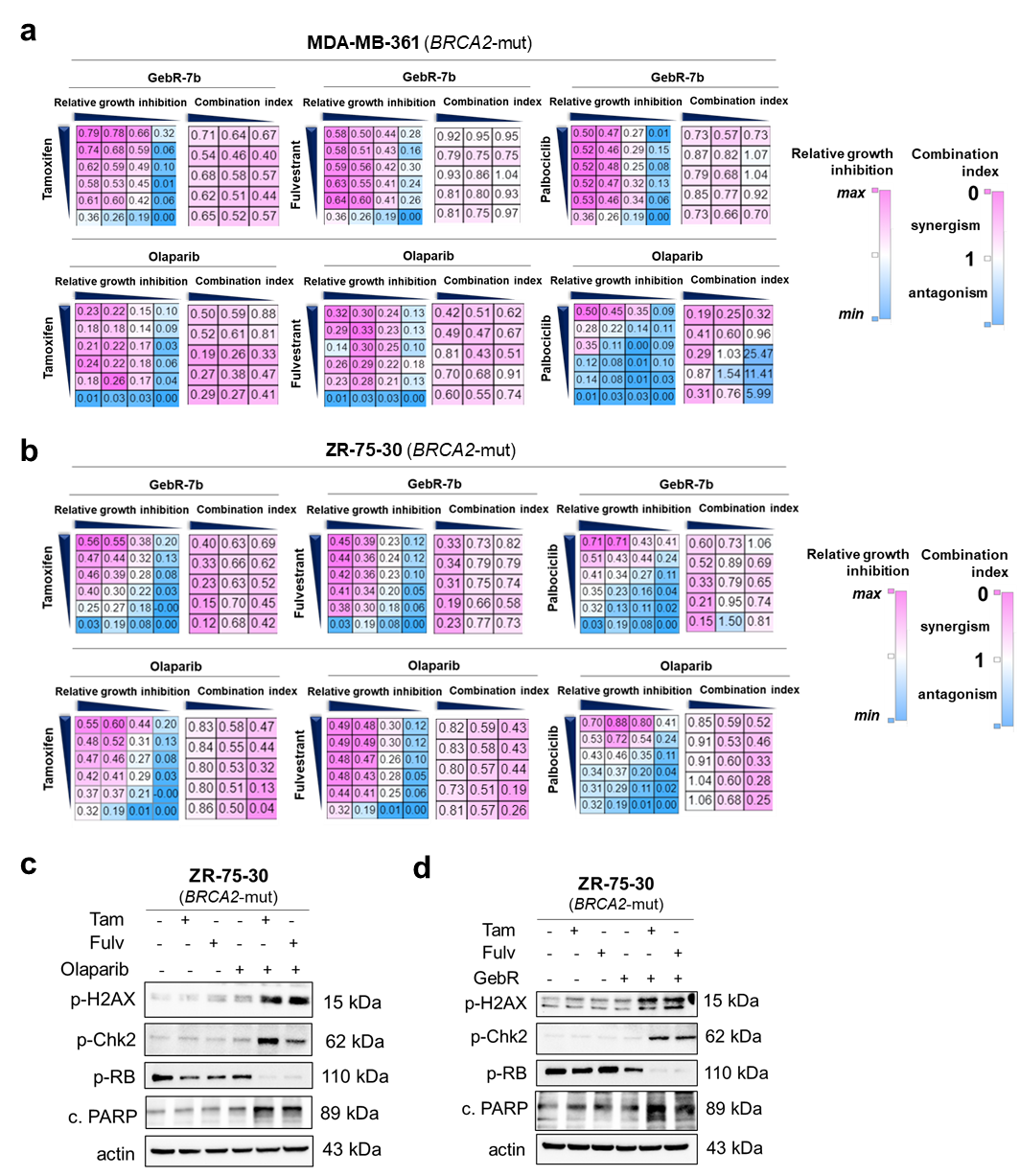


**Supplementary Figure 9. Inhibition of PDE4D or PARP overcomes SOC resistance in *BRCA2*-mut cells. a, b** Heatmaps of relative growth inhibition and combination indices in MDA-MB-361 (c) or ZR-75-30 (d) cells treated with different SOC therapies (for MDA-MB-361: tamoxifen: 0.5, 1, 1.5, 2, 3.5 µM; fulvestrant: 0.5, 1, 2.5, 5, 10 µM; palbociclib: 2.5, 5, 10, 20, 30 µM. For ZR-75-30: tamoxifen: 2, 3, 4, 5, 7.5 µM; fulvestrant: 5, 10, 15, 25, 35 µM; palbociclib: 15, 20, 25, 30, 35 µM) and GebR-7b (for MDA-MB-361: 50, 75, 100 µg/ml; for ZR-75-30: 5, 10, 20 µg/ml), or olaparib (for MDA-MB-361: 10, 25, 50 µM; for ZR-75-30: 10, 20, 30 µM). **c, d** Western blot analysis of the markers in ZR-75-30 cells treated with tamoxifen/fulvestrant with or without olaparib (c) or GebR-7b (d) for 24 hours. Experiment is repeated twice with similar results. Source data for this figure are provided as a Source Data file.


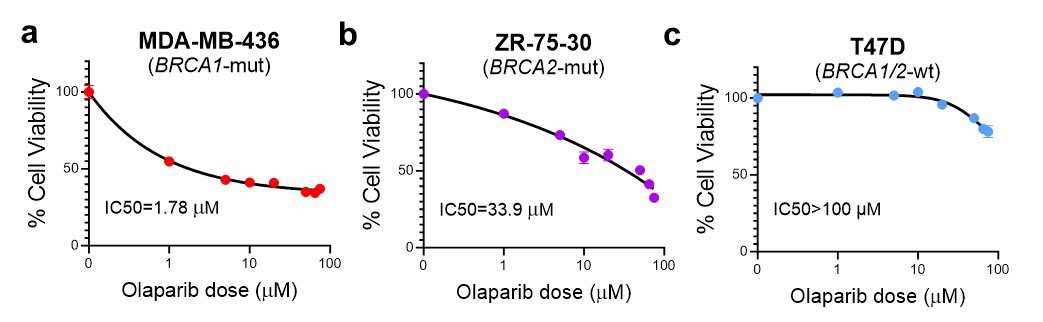


**Supplementary Figure 10. Olaparib response of the *BRCA1/2*-wt versus *BRCA1/2*-mut cell lines. a-c** Percentage cell viability in MDA-MB-436 (*BRCA1*-mut) (a), ZR-75-30 *(BRCA2*-mut) (b) and T47D (*BRCA1/2*-wt) (c) cells upon treatment with olaparib for 3 days (n=3, 4). Data are presented as mean values ± standard deviation (SD). Source data for this figure are provided as a Source Data file.


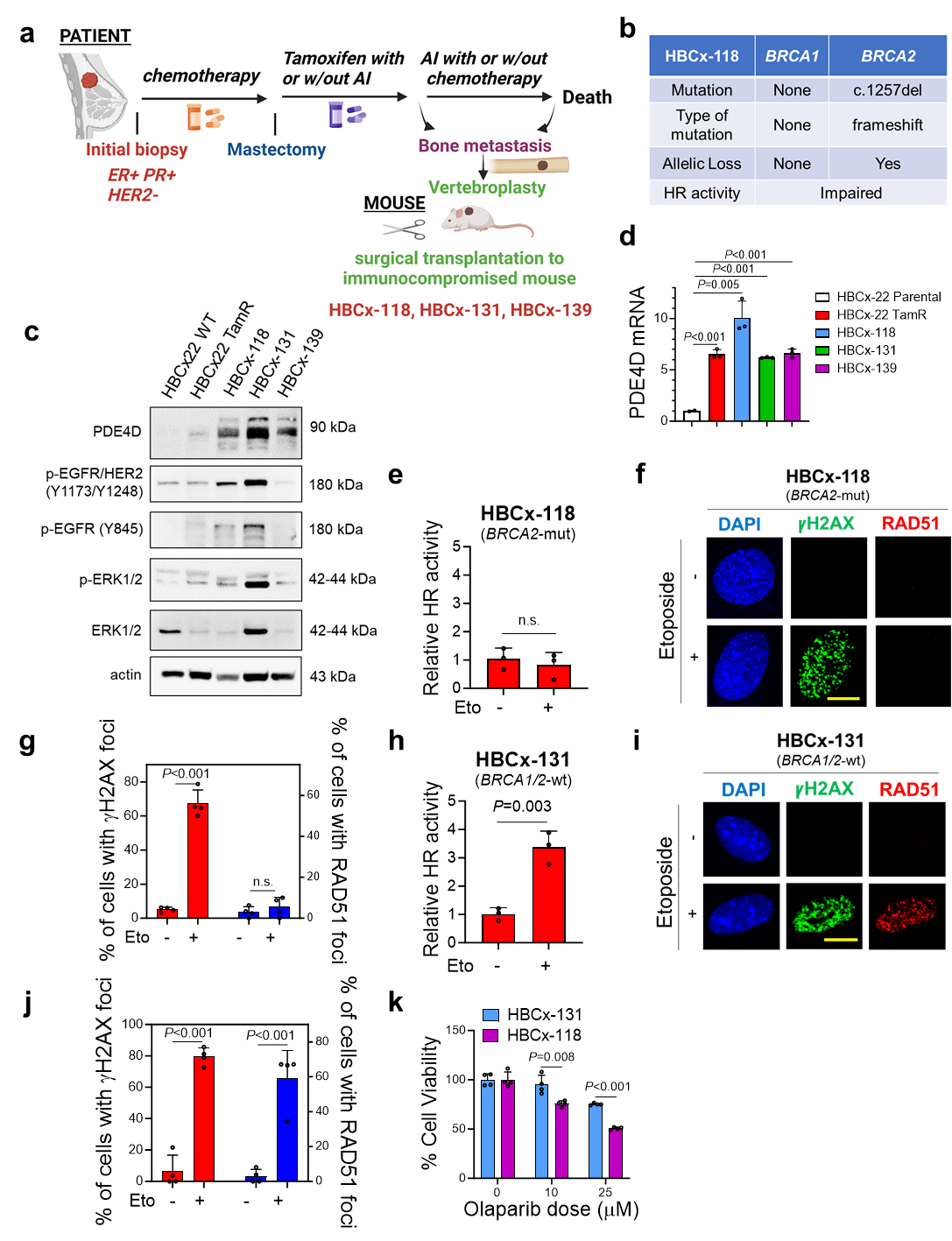


**Supplementary Figure 11. Endocrine resistant ER+ PDXs express PDE4D and EGFR signaling downstream and the *BRCA2*-mut PDX, HBCx-118 is homologous recombination (HR) deficient. a** Treatment scheme of the patients whose endocrine resistant ER+ tumors were used to develop PDX models, HBCx-118, HBCx-131 and HBCx-139. The figure is created with BioRender.com. **b** Table of the *BRCA1* and *BRCA2* mutations, mutation types, the allelic changes and the HR activity of HBCx-118 PDX. **c** Western blot analysis of PDE4D, EGFR and downstream pathway markers in separate endocrine resistant ER+ PDX tumors, collected from different mice bearing tumors of a different model. **d** qRT-PCR analysis of PDE4D in parental and endocrine resistant ER+ PDX tumors (n=2, 3). **e, h** Relative homologous recombination (HR) activity in HBCx-118 (e) and HBCx-131 (h) PDX cells upon treatment with etoposide, showing HR deficiency in the *BRCA2*-mut HBCx-118 model (n=3). **f, i** IF staining of γ-H2AX (S139) (green) and RAD51 (red) in HBCx-118 (*BRCA2*-mut) (f) and HBCx-131 (*BRCA1/2* wt) (i) cells upon treatment with etoposide for 4 hours. **g, j** The quantification of γ-H2AX positive cells and those that are RAD51 positive in HBCx-118 (g) and HBCx-131 (j) PDXs (n=4 different areas, with at least 100 cells per area). DAPI (blue) was used to stain the nucleus. **k** Percentage cell viability in HBCx-131 (*BRCA1/2* wt) versus HBCx-118 (*BRCA2*-mut) PDX cells upon treatment with olaparib for 3 days (n=4). Data are presented as mean values ± standard deviation (SD). *P*-values were calculated with the unpaired, two-tailed Student’s t test. n.s., not significant (*P*>0.05). Source data for this figure are provided as a Source Data file.

**
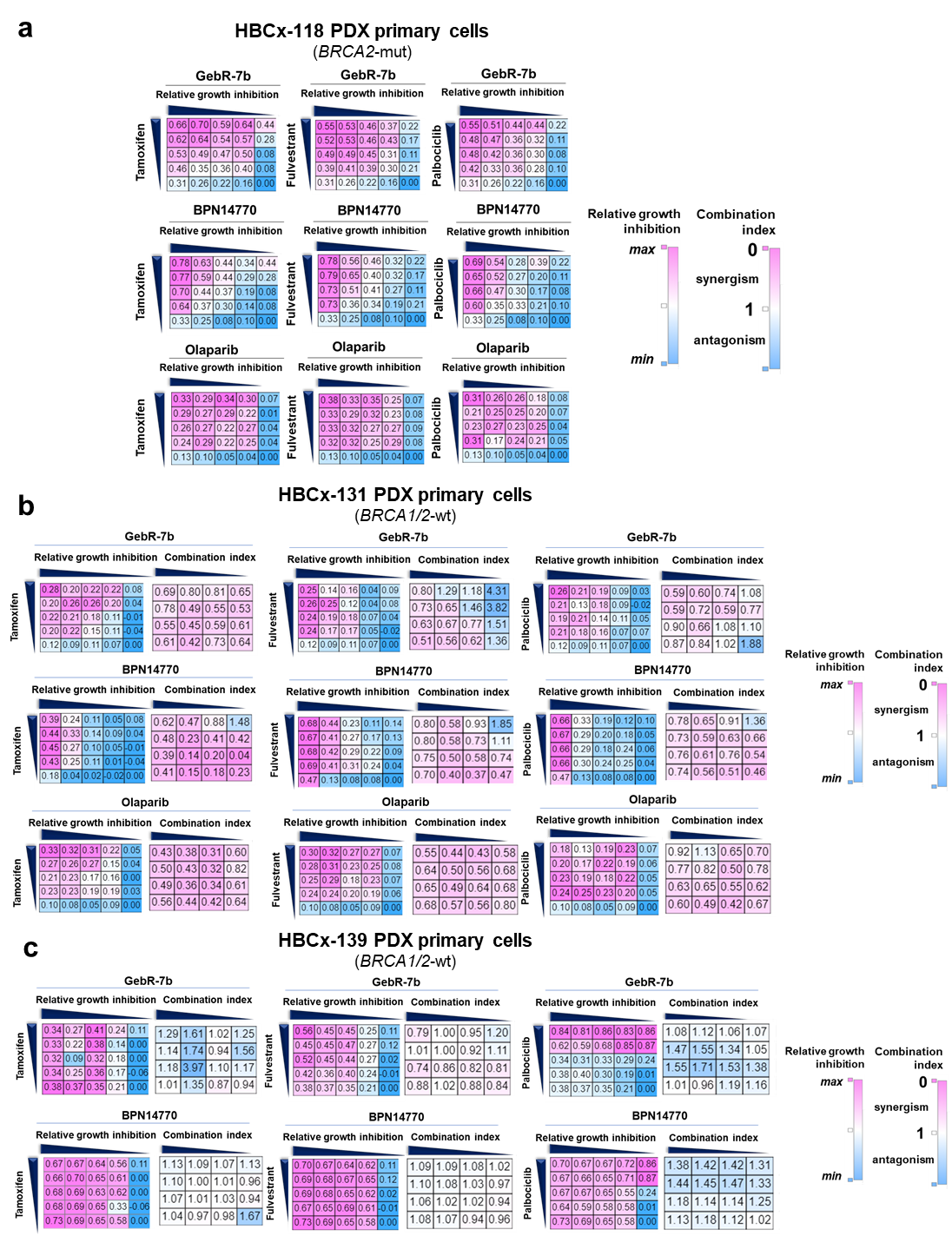
**

**Supplementary Figure 12. Combining PDE4D or PARP inhibitor with SOC therapies in different endocrine resistant ER+ primary PDX cells.** **a, b** Heatmaps of relative growth inhibition (left panel) and combination indices (right panel) in primary cell cultures of HBCx-118 (A) and HBCX-131 (B) models treated with the combination of different SOC therapies (tamoxifen: 2, 3, 4, 4.5 µM; fulvestrant: 1, 5, 10, 20 µM; palbociclib: 0.5, 1, 1.5, 2.5 µM) and GebR-7b (30, 40, 50, 60 µg/mL) or BPN14770 (35, 40, 45, 50 µg/mL) or olaparib (2.5, 5, 7.5, 10 µM). **c** Heatmaps of relative growth inhibition (left panel) and combination indices (right panel) in primary cell cultures of HBCX-139 model treated with the combination of different SOC therapies (tamoxifen: 1, 2, 3, 4 µM; fulvestrant: 1, 5, 10, 20 µM; palbociclib: 0.1, 0.5, 1, 2.5 µM) and GebR-7b or BPN14770 (60, 70, 80, 90 µg/mL). Source data for this figure are provided as a Source Data file.

**
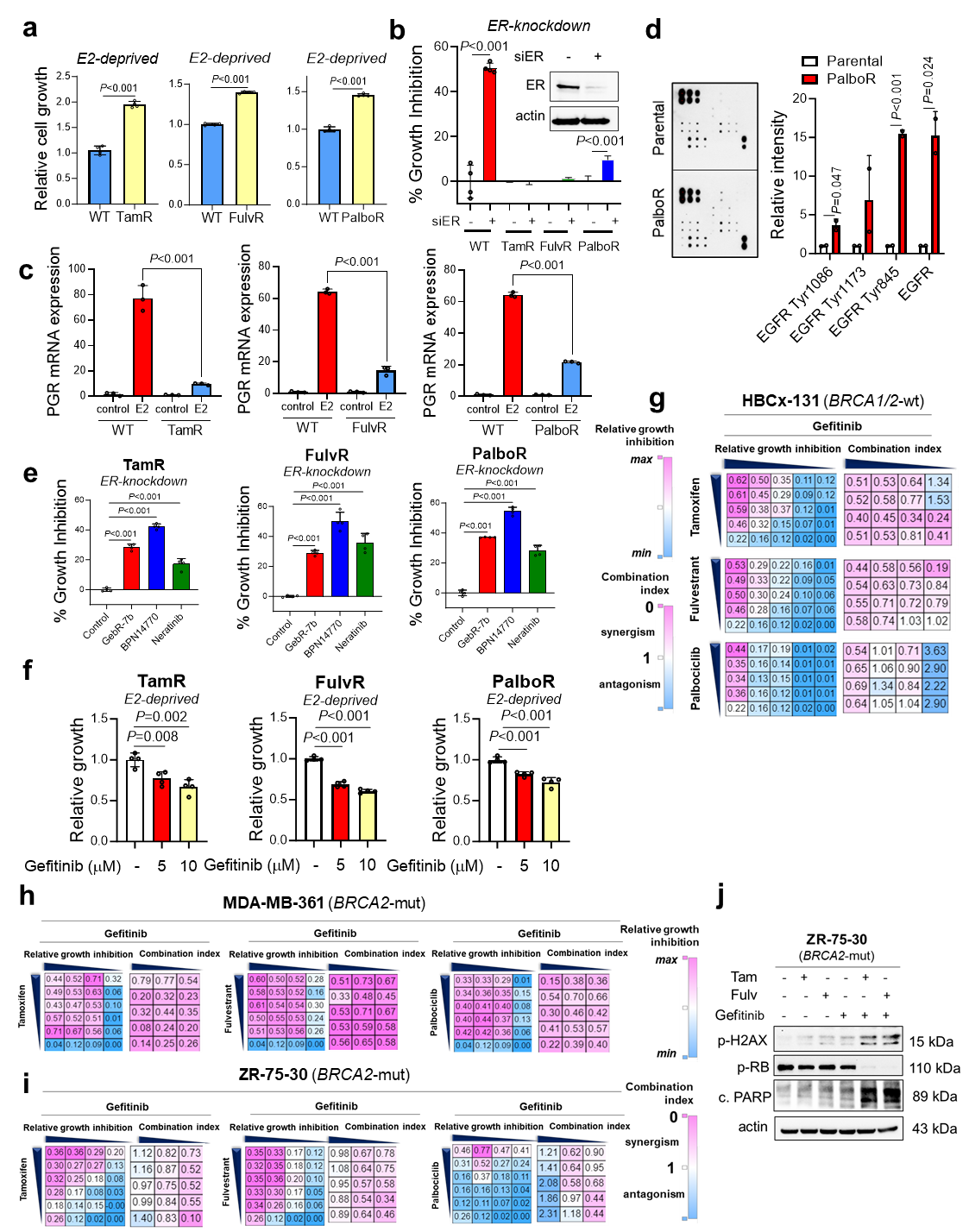
**

**Supplementary Figure 13. Characterization of ER independence and EGFR dependence of SOC resistant cells, targeting EGFR to overcome SOC resistance in *BRCA1/2*-wt and *BRCA2*-mut cells and western blot analyses of DNA damage, G1 arrest and apoptosis markers upon EGFR inhibition. a** Relative growth of parental vs. SOC resistant MCF-7 cells in ER-deprived media (n=4). **b** Percentage growth inhibition in T47D parental vs. SOC resistant cells upon ER knockdown with siRNA for 72 hours. Western blot validation of ER knockdown is shown on the graph (n=4). **c** qRT-PCR analyses of the ER target, PGR in parental vs. SOC resistant cells stimulated with E2 for 24 hours (n=3). **d** EGFR array in T47D parental vs. PalboR cells (n=2). **e** Percentage growth inhibition in SOC resistant cells upon ER knockdown with siRNA and under treatment with GebR-7b, BPN14770 or neratinib (n=4). **f** Relative growth of SOC resistant MCF-7 cells under E2-deprived media, treated with increasing doses of gefitinib for 3 days (n=4). **g** Heatmaps of relative growth inhibition (left panel) and combination indices (right panel) in primary cell cultures of HBCx-131 models treated with the combination of different SOC therapies (tamoxifen: 2, 3, 4, 4.5 µM; fulvestrant: 1, 5, 10, 20 µM; palbociclib: 0.5, 1, 1.5, 2.5 µM) and gefitinib (1, 5, 7,5 10 µM). **h** Heatmaps of relative growth inhibition (left panel) and combination indices (right panel) in MDA-MB-361 cells treated with different SOC therapies (tamoxifen: 0.5, 1, 1.5, 2, 3.5 µM; fulvestrant: 0.5, 1, 2.5, 5, 10 µM; palbociclib: 2.5, 5, 10, 20, 30 µM) and GebR-7b (50, 75, 100 µg/ml), gefitinib (0.1, 0.2, 0.5 µM) or olaparib (10, 25, 50 µM). **i** Heatmaps of relative growth inhibition (left panel) and combination indices (right panel) in ZR-75-30 cells treated with different SOC therapies (tamoxifen: 2, 3, 4, 5, 7.5 µM; fulvestrant: 5, 10, 15, 25, 35 µM; palbociclib: 15, 20, 25, 30, 35 µM) and GebR-7b (5, 10, 20 µg/ml), gefitinib (0.1, 0.5, 1 µM) or olaparib (10, 20, 30 µM). **j** Western blot analysis of the markers in ZR-75-30 cells treated with tamoxifen or fulvestrant in combination with gefitinib for 24 hours. Experiment is repeated twice with similar results. Data are presented as mean values ± standard deviation (SD). *P*-values were calculated with the unpaired, two-tailed Student’s t test. Source data for this figure are provided as a Source Data file.


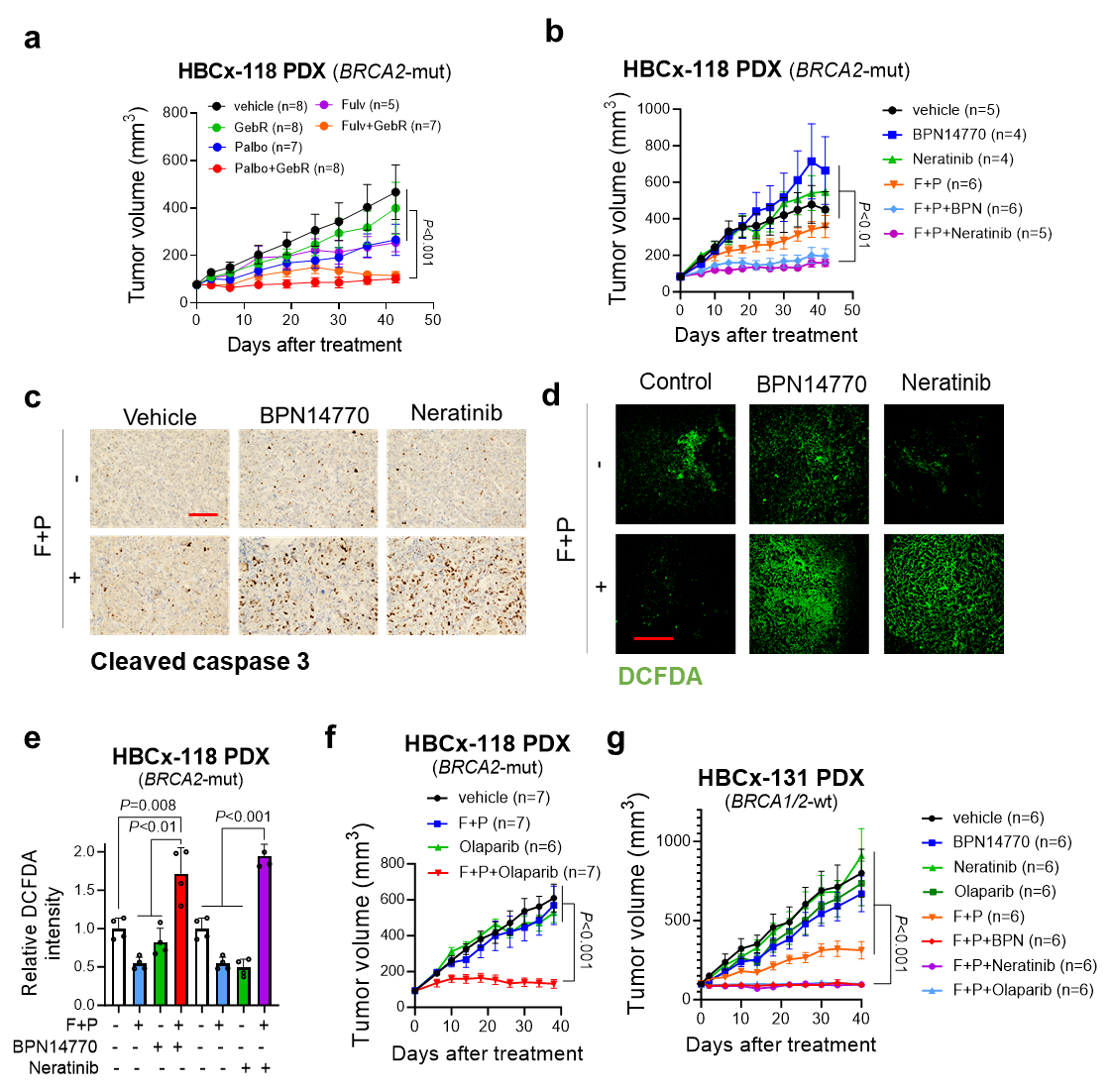


**Supplementary Figure 14. Absolute tumor volume graphs of the PDX experiments and cleaved caspase 3 and ROS staining in PDX tumors. a** Tumor volume in mm^3^ over time in HBCx-118 PDXs upon treatment with fulvestrant (35 mg/kg, subcutaneous) or palbociclib (35 mg/kg, oral gavage) in combination with PDE4D inhibitor, GebR-7b (3 µg/kg, intraperitoneal) (n = 5-8). **b** Tumor volume in mm^3^ over time in HBCx-118 PDXs upon treatment with fulvestrant (20 mg/kg, subcutaneous) plus palbociclib (20 mg/kg, oral gavage) in combination with PDE4D inhibitor, BPN14770 (0.75 mg/kg, oral gavage) or the pan-HER inhibitor, neratinib (15 mg/kg, oral gavage) (n = 4-6). **c** Cleaved caspase 3 staining (brown) in HBCx-118 tumors from b. Scale bar=100 µm. **d** Representative images of the HBCx-118 PDX tumors stained with the ROS marker, DCFDA (green), collected the end of the experiment from b. Scale bar=500 µm. **e** Quantification of DCFDA staining in d (n=4). **f** Tumor volume in mm^3^ over time in HBCx-118 PDXs upon treatment with fulvestrant (F, 25 mg/kg, subcutaneous) + palbociclib (P, 25 mg/kg, oral gavage) in combination with PARP inhibitor, olaparib (35 mg/kg, oral gavage) (n = 6-7). **g** Tumor volume in mm^3^ over time in HBCx-131 PDXs upon treatment with fulvestrant (15 mg/kg, subcutaneous) plus palbociclib (15 mg/kg, oral gavage) in combination with PDE4D inhibitor, BPN14770 (0.75 mg/kg, oral gavage) or neratinib (15 mg/kg, oral gavage) or olaparib (35 mg/kg, oral gavage) (n = 6). Data for the bar graphs are presented as mean values ±SD, while data for the tumor volume graph are presented as mean values ± standard error of the mean (SEM). *P*-values for the bar graphs were calculated with the unpaired, two-tailed Student’s t test. Significance for the tumor volume graphs was calculated with two-way ANOVA. Source data for this figure are provided as a Source Data file.

**
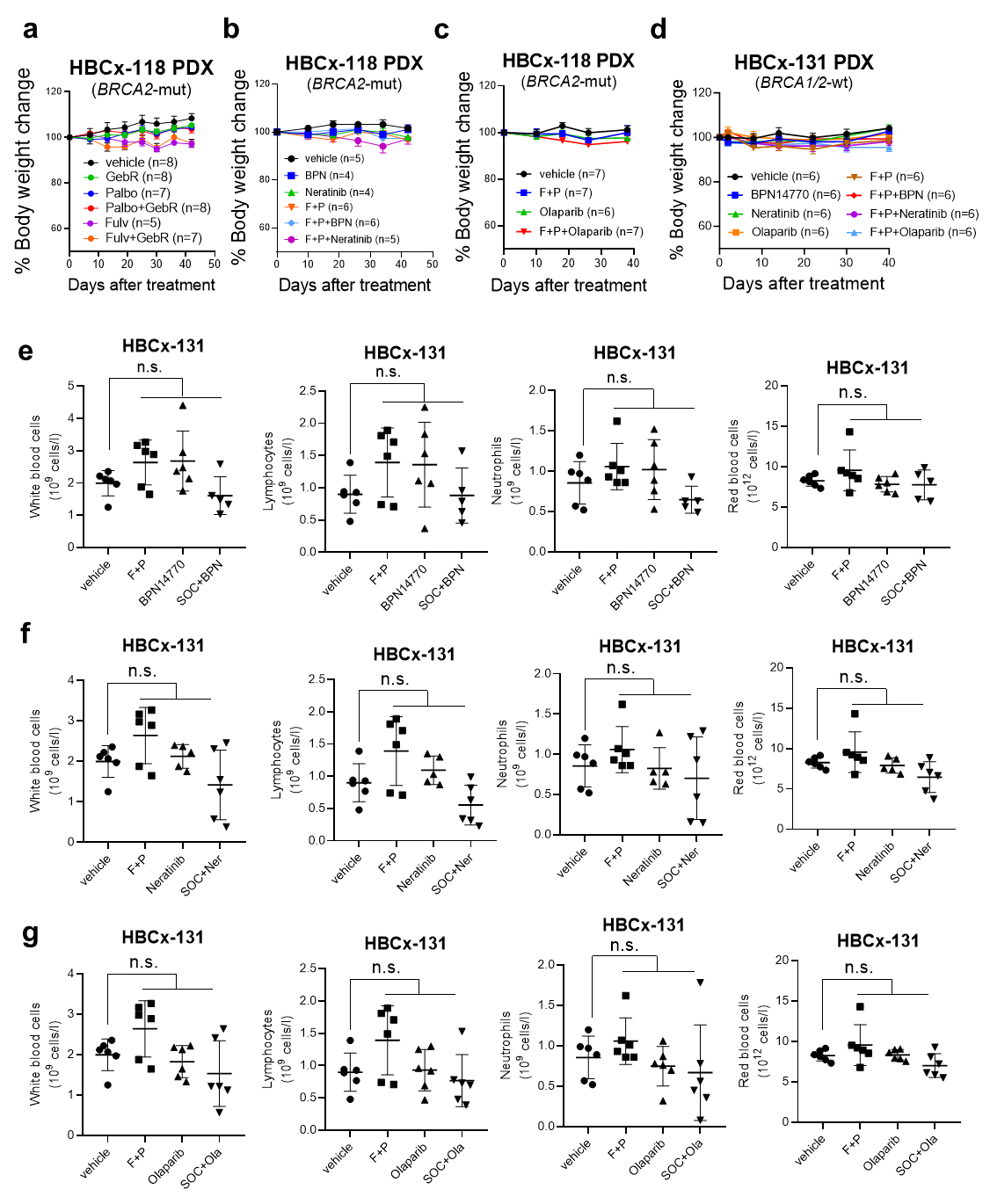
**

**Supplementary Figure 15. Body weight changes and blood counts in PDXs treated with SOC in combination with inhibitors targeting PDE4D, EGFR or PARP. a** Percent body weight changes in HBCx-118 PDXs upon treatment with fulvestrant (35 mg/kg, subcutaneous) or palbociclib (35 mg/kg, oral gavage) in combination with PDE4D inhibitor, GebR-7b (3 µg/kg, intraperitoneal) (n = 5-8). **b** Percent body weight change in HBCx-118 PDXs upon treatment with fulvestrant (20 mg/kg, subcutaneous) plus palbociclib (20 mg/kg, oral gavage) in combination with PDE4D inhibitor, BPN14770 (0.75 mg/kg, oral gavage) or the pan-HER inhibitor, neratinib (15 mg/kg, oral gavage) (n=4-6). **c** Percent body weight change in HBCx-118 PDXs upon treatment with fulvestrant (F, 25 mg/kg, subcutaneous) + palbociclib (P, 25 mg/kg, oral gavage) in combination with PARP inhibitor, olaparib (35 mg/kg, oral gavage) (n = 6-7). **d** Percent body weight change in HBCx-131 PDXs upon treatment with fulvestrant (15 mg/kg, subcutaneous) plus palbociclib (15 mg/kg, oral gavage) in combination with PDE4D inhibitor, BPN14770 (0.75 mg/kg, oral gavage) or neratinib (15 mg/kg, oral gavage) or olaparib (35 mg/kg, oral gavage) (n = 6). **e** Blood counts form mice in d at the end of treatment (n=5, 6). Data are presented as mean values ± standard deviation (SD). *P*-values were calculated with one-way ANOVA. n.s., not significant (*P*>0.05). Source data for this figure are provided as a Source Data file.

**SUPPLEMENTARY TABLES**

**Supplementary Data 1.** List of genes within the SOC sensitivity signature along with the z-score values comparing treatment vs. untreated control, downloaded from Connectivity Map database. This table is provided as an Excel table.

**Supplementary Table 1.** Characteristics of the endocrine resistant PDXs, HBCx-118, HBCx-131, HBCx-139.

| **Xenograft ID** | **PDX** | | | **Treatment before vertebroplasty** |
| --- | --- | --- | --- | --- |
|  | **Mutations** | **Gene Amplification** | **Homozygous deletion** |  |
| **HBCx-118** | *BRCA2, AKT1* |  | *FGFR4* | FEC, Vinorelbine, Tamoxifen, Letrozole, Paclitaxel, Capecitabine |
| **HBCx-131** |  | *CCND1* | *CDKN2A/B* | Cyclophospamide+Epirubicine+Docetaxel, AC, FUN,  Tamoxifen, Anastrozole, Letrozole |
| **HBCx-139** | *PIK3CA* | *PAK1* | *CCDN1, CCNB1, CDK7, CCNE2* | FEC, Tamoxifen+Triptorelin, Docetaxel, Capecitabine, Cyclophosphamide, Paclitaxel, Letrozole, Exemestane+Everolimus, Doxorubicin, Fulvestrant |

**Supplementary Table 2.** Target sequences for the shRNAs.

| **Gene Name** | **NCBI Gene ID** | **Company** | **Catalog number** | **sequence** |
| --- | --- | --- | --- | --- |
| PARP1 | 142 | Sigma | TRCN0000007932 | CGACCTGATCTGGAACATCAA |
| PARP1 | 142 | Sigma | TRCN0000007929 | GCAGCTTCATAACCGAAGATT |

**Supplementary Table 3.** Sequences of siRNAs, qRT-PCR primers and CHIP qPCR primers.

| ***Sequences of siRNAs used*** | | | | | | | | | |  |
| --- | --- | --- | --- | --- | --- | --- | --- | --- | --- | --- |
| **Gene Name** | **NCBI Gene ID** | | | **Company** | | | **Catalog number** | | **sequence** |  |
| Jun | 3725 | | | Dharmacon | | | D-003268-22-0002 | | AAGUCAUGAACCACGUUAA |  |
| PDE4D | 5144 | | | Dharmacon | | | D-004757-02-0002 | | GAACUUGCCUUGAUGUACA |  |
| ESR1 | 2099 | | | Dharmacon | | | D-003401-02-0002 | | CAUGAGAGCUGCCAACCUU |  |
| PARP1 | 142 | | | Dharmacon | | | D-006656-03-0002 | | GCAACAAACUGGAACAGAU |  |
| ***Sequences of qRT-PCR primers*** | | | | | | | | | | |
| **Gene Name** | | **NCBI Gene ID** | | | |  | | **Primer sequence** | | |
| ACTB | | 60 | | | | Forward | | 5’-CCAACCGCGAGAAGATGA-3’ | | |
|  |  |  |  |  |  | Reverse | | 5’-CCAGAGGCGTACAGGGATAG-3’ | | |
| HPRT | | 3251 | | | | Forward | | 5’-TGACCTTGATTTATTTTGCATACC-3’ | | |
|  |  |  |  |  |  | Reverse | | 5’-CGAGCAAGACGTTCAGTCCT-3’ | | |
| PDE4D3 | | 1612 | | | | Forward | | 5’-GCGAACATGATGCACGTGAA-3’ | | |
|  |  |  |  |  |  | Reverse | | 5’-TGGCCAAGACCTGAGCAAAT-3’ | | |
| PDE4D_Pan | | 1612 | | | | Forward | | 5’-ATTGCCAGTGATATACACGGA-3’ | | |
|  |  |  |  |  |  | Reverse | | 5’-GTTTCGTACAGTTCGCAGAC-3’ | | |
| JUN | | 5655 | | | | Forward | | 5’-TCAGACAGTGCCCGAGATG-3’ | | |
|  |  |  |  |  |  | Reverse | | 5’-CTGCTGCGTTAGCATGAGTT-3’ | | |
| PGR | | 317 | | | | Forward | | 5’-CAGGTCTACCCGCCCTATCT-3’ | | |
|  |  |  |  |  |  | Reverse | | 5’-TAGTTGTGCTGCCCTTCCAT-3’ | | |
| ***Sequences of c-Jun ChIP qPCR primers*** | | | | | | | | | | |
| **Promoter/Gene name** | | | **PDE4D isoform** | | |  | | **Primer sequence** | | |
| P1 | | | PDE4D5 | | | Forward | | 5’- GTCTGCACTTCTAGTGTCCACAT-3’ | | |
|  |  |  |  |  |  | Reverse | | 5’- ACTAATGCCCCTCCAGAAGGA-3’ | | |
| P2 | | | PDE4D7 | | | Forward | | 5’- TGCCTCAGCGACTTTATGCTT-3’ | | |
|  |  |  |  |  |  | Reverse | | 5’- TGTCGGAGGGGAAAAGGACA-3’ | | |
| P3 | | | PDE4D3 | | | Forward | | 5’- GCTGCGAGGATTCCAAAGGT-3’ | | |
|  |  |  |  |  |  | Reverse | | 5’- AAACACAATGGCAACAGGCTC-3’ | | |
| P6 | | | PDE4D4 | | | Forward | | 5’- CAAGCTCTGAGAACCAGCCA-3’ | | |
|  |  |  |  |  |  | Reverse | | 5’- CTCTTGATCCTTCCCCTCGC-3’ | | |
| P7 | | | PDE4D8 | | | Forward | | 5’- CTCAGTGGTGAGGACCGATG-3’ | | |
|  |  |  |  |  |  | Reverse | | 5’- CTCAAGGAGATGCTGCGTCA-3’ | | |
| P8 | | | PDE4D9 | | | Forward | | 5’- AGATAGGCAGGCTGGTCAAC-3’ | | |
|  |  |  |  |  |  | Reverse | | 5’- GTGGCTGCACGTATTAAGGC-3’ | | |
| ERCC1 | | |  | | | Forward | | 5’- GATGTCCCTTCCTCCAGGAA-3’ | | |
|  |  |  |  |  |  | Reverse | | 5’- AATGGGCAGGTCTGGGATGG-3’ | | |
| BCL2L1 | | |  | | | Forward | | 5’- TACTTCGTCTGTCTCCCTCACT-3’ | | |
|  |  |  |  |  |  | Reverse | | 5’- AGCACCCGTTCCTTCCCTTAT-3’ | | |
| ***Sequences of ERα ChIP qPCR primers*** | | | | | | | | | | |
| **Promoter/Gene name** | | | **PDE4D isoform** | |  | | **Primer sequence** | | | |
| P3 | | | PDE4D3 | | Forward | | 5’- TAGCACTGCGTTAAAGTGACC-3’ | | | |
|  |  |  |  |  | Reverse | | 5’- AAATCTAACCGCCTTGAGTGT-3’ | | | |
| P7 | | | PDE4D8 | | Forward | | 5’- ACACCAGAAGGGCAGATTGG-3’ | | | |
|  |  |  |  |  | Reverse | | 5’- TGAACAGCAGCAACCTTGGA-3’ | | | |
| P8 | | | PDE4D9 | | Forward | | 5’- AGGGGAGAGGGACGATTTCT-3’ | | | |
|  |  |  |  |  | Reverse | | 5’- AATTAGCCTGTGTTCTACCGT-3’ | | | |
| GREB1 | | |  | | Forward | | 5’- AGCAGTGAAAAAAAGTGTGGCAACTGGG-3’ | | | |
|  |  |  |  |  | Reverse | | 5’- GACCCACAGAAATGAAAAGGCAGCAAACT-3’ | | | |
| PGR | | |  | | Forward | | 5’- GTAATCAAATCTGTGGCACACC-3’ | | | |
|  |  |  |  |  | Reverse | | 5’- GTCCTCAAAAACTCAATTTCATAAGT-3’ | | | |

**Supplementary Table 4.** List of antibodies used in Western blot (WB), immunofluorescence (IF), immunoprecipitation (IP) and chromatin immunoprecipitation (ChIP) experiments.

| ***List of antibodies used in Western blot (WB), immunofluorescence (IF), immunoprecipitation (IP) and chromatin immunoprecipitation (ChIP) experiments.*** | | | | | |
| --- | --- | --- | --- | --- | --- |
| **Antibody** | **Provider** | **Catalog number** | **WB dilution** | **IF dilution** | **IP/ChIP dilution** |
| Alexa Fluor® 488 anti-mouse | Life Technologies | A-11001 | - | 1:1000 | - |
| Alexa Fluor® 647 anti-rabbit | Life Technologies | A-31573 | - | 1:1000 | - |
| Beta-actin | MP Biomedicals | 691001 | 1:10000 | - | - |
| GAPDH | Santa Cruz | sc-47724 | 1:10000 | - | - |
| PDE4D | ProteinTech | 12918-1-AP | 1:1000 | - | - |
| FEN1 | ProteinTech | 14768-1-AP | 1:1000 | - | - |
| XRCC1 | Santa Cruz | sc-56254 | 1:1000 | - | - |
| RAD51 | Abcam | ab133534 | 1:1000 | 1:150 | - |
| BRCA1 | ProteinTech | 22362-1-AP | 1:1000 | - | - |
| BRCA2 | Cell Signaling | 10741 | 1:1000 |  |  |
| PARP1 | BD Biosciences | 556494 | 1:1000 | - | - |
| p-RB | Cell Signaling | 8516 | 1:1000 | - | - |
| HPF1 | Cell Signaling | 90876 | 1:1000 | - | - |
| Acetyl-Histone H3 (Lys9) | Cell Signaling | 9649 | 1:1000 | - | - |
| Histone H3 | Cell Signaling | 4499 | 1:1000 | - | - |
| H3S10 ADPr | BioRad | HCA357 | 1:1000 | - | - |
| PAR/pADPr | R&D systems | 4335-MC-100 | - | - | 1:100 (IP) |
| Poly/Mono-ADP Ribose | Cell Signaling | 9649 | 1:1000 | - | - |
| Alpha-tubulin | Santa Cruz | sc-32293 | 1:1000 | - | - |
| ER | Santa Cruz | sc-8002 | 1:1000 | - | - |
| p-H2AX (S139) | Santa Cruz | sc-517348 | 1:1000 | 1:500 | - |
| p-Chk2 (T68) | Cell Signaling | 2197 | 1:1000 | - | - |
| p-Chk1 (S345) | Cell Signaling | 2348 | 1:1000 | - | - |
| p-PKA (Thr197) | Cell Signaling | 4781 | 1:1000 | - | - |
| p-CREB (S133) | Cell Signaling | 9198 | 1:1000 | - | - |
| p-ER (S118) | Cell Signaling | 2511 | 1:1000 | - | - |
| p-ERK1/2 (T202/Y204) | Cell Signaling | 4376 | 1:1000 | - | - |
| p-EGFR (Tyr845) | Sigma | 07-820 | 1:1000 | - | - |
| p-HER2 (Y1248)-EGFR (Y1173) | Cell Signaling | 2244 | 1:1000 | - | - |
| p-AKT (S473) | Cell Signaling | 4058 | 1:1000 | - | - |
| AKT | Cell Signaling | 9272 | 1:1000 | - | - |
| Cyclin D1 | Cell Signaling | 2922 | 1:1000 | - | - |
| Cleaved PARP | Cell Signaling | 5625 | 1:1000 | - | - |
| CDK4 | Abcam | ab68266 | 1:1000 | - | - |
| ERK1/2 | Cell Signaling | 4695 | 1:1000 | - | - |
| c-Myc | Santa Cruz | sc-40 | 1:1000 | - | - |
| c-Jun | Cell Signaling | 9165 | 1:1000 | - | 1:50 (ChIP) |
| ER | Active Motif | 61035 | - | - | 1:100 (ChIP) |
| COXIV | ProteinTech | 11242-1-AP | 1:1000 | - | 1:100 (IP) |

**SUPPLEMENTARY REFERENCES**

1 Foucquier, J. & Guedj, M. Analysis of drug combinations: current methodological landscape. *Pharmacol Res Perspect*.**3**, e00149 (2015).

2 Assidicky, R. et al. Targeting HIF1-alpha/miR-326/ITGA5 axis potentiates chemotherapy response in triple-negative breast cancer. *Breast Cancer Res Treat*.**193**, 331-348 (2022).

3 Saatci, O. et al. Targeting TACC3 represents a novel vulnerability in highly aggressive breast cancers with centrosome amplification. *Cell Death Differ*.**30**, 1305-1319 (2023).

4 Akbulut, O. et al. A Highly Potent TACC3 Inhibitor as a Novel Anticancer Drug Candidate. *Mol Cancer Ther*.**19**, 1243-1254 (2020).

5 Saatci, O. et al. Targeting lysyl oxidase (LOX) overcomes chemotherapy resistance in triple negative breast cancer. *Nat Commun*.**11**, 2416 (2020).

6 Wang, Q. & Zou, M. H. Measurement of Reactive Oxygen Species (ROS) and Mitochondrial ROS in AMPK Knockout Mice Blood Vessels. *Methods Mol Biol*.**1732**, 507-517 (2018).
