# Supplementary material for: Toxic PARP trapping upon cAMP-induced DNA damage reinstates the efficacy of endocrine therapy and CDK4/6 inhibitors in treatment-refractory ER+ breast cancer
Source: Nat Commun. 2023 Nov 2;14:6997. doi: 10.1038/s41467-023-42736-y (PMC10620179; doi:10.1038/s41467-023-42736-y)
Supplement: Supplementary file 3 — Description of Additional Supplementary Files [file 41467_2023_42736_MOESM3_ESM.pdf]

Description of Additional Supplementary Files

File Name: Supplementary Data 1
Description: List of genes within the SOC sensitivity signature along with the z-score values comparing treatment vs. untreated control, downloaded from Connectivity Map database.
