# Supplementary material for: Toxic PARP trapping upon cAMP-induced DNA damage reinstates the efficacy of endocrine therapy and CDK4/6 inhibitors in treatment-refractory ER+ breast cancer
Source: Nat Commun. 2023 Nov 2;14:6997. doi: 10.1038/s41467-023-42736-y (PMC10620179; doi:10.1038/s41467-023-42736-y)
Supplement: Supplementary file 5 — Reporting Summary [file 41467_2023_42736_MOESM5_ESM.pdf]

nature portfolio

Corresponding author(s): Ozgur Sahin

Last updated by author(s): Sep 14, 2023

Reporting Summary

Nature Portfolio wishes to improve the reproducibility of the work that we publish. This form provides structure for consistency and transparency in reporting. For further information on Nature Portfolio policies, see our Editorial Policies and the Editorial Policy Checklist.

Statistics

For all statistical analyses, confirm that the following items are present in the figure legend, table legend, main text, or Methods section.

n/a	Confirmed	
☐	☒	The exact sample size (*n*) for each experimental group/condition, given as a discrete number and unit of measurement
☐	☒	A statement on whether measurements were taken from distinct samples or whether the same sample was measured repeatedly
☐	☒	The statistical test(s) used AND whether they are one- or two-sided<br>*Only common tests should be described solely by name; describe more complex techniques in the Methods section.*
☒	☐	A description of all covariates tested
☒	☐	A description of any assumptions or corrections, such as tests of normality and adjustment for multiple comparisons
☐	☒	A full description of the statistical parameters including central tendency (e.g. means) or other basic estimates (e.g. regression coefficient) AND variation (e.g. standard deviation) or associated estimates of uncertainty (e.g. confidence intervals)
☐	☒	For null hypothesis testing, the test statistic (e.g. *F*, *t*, *r*) with confidence intervals, effect sizes, degrees of freedom and *P* value noted<br>*Give P values as exact values whenever suitable.*
☒	☐	For Bayesian analysis, information on the choice of priors and Markov chain Monte Carlo settings
☒	☐	For hierarchical and complex designs, identification of the appropriate level for tests and full reporting of outcomes
☒	☐	Estimates of effect sizes (e.g. Cohen's *d*, Pearson's *r*), indicating how they were calculated

Our web collection on statistics for biologists contains articles on many of the points above.

Software and code

Policy information about availability of computer code

Data collection	CFX Manager Software version 3.0 (Biorad), QuantStudio software 6, SoftMax Pro Software version 7.1 (Molecular Devices), Image Lab Software version 6.0.0 (Biorad), iBright 1500 version 1.8 (Invitrogen), NIS-Elements 5.02.00 (Nikon), Zeiss 880 LSM NLO, BD LSR II flow cytometry (BD), FACSDiva version 7 (BD), GSEA version 4.2.3, VetScan HM5 v2.61.
Data analysis	Excel 2016 (Microsoft), GraphPad Prism software version 8, ImageJ version 2 (FIJI), SPSS Software version 22 (IBM), FlowJo version 10.8.1, FACSDiva version 7, De Novo Software: FCS Express version 6.

For manuscripts utilizing custom algorithms or software that are central to the research but not yet described in published literature, software must be made available to editors and reviewers. We strongly encourage code deposition in a community repository (e.g. GitHub). See the Nature Portfolio guidelines for submitting code & software for further information.

Data

Policy information about availability of data

All manuscripts must include a data availability statement. This statement should provide the following information, where applicable:

- Accession codes, unique identifiers, or web links for publicly available datasets
- A description of any restrictions on data availability
- For clinical datasets or third party data, please ensure that the statement adheres to our policy

Source data are provided with this paper. Biological materials are available from the corresponding author upon reasonable request. Data presented on Fig. 1a was generated by analyzing the Connectivity Map Database. Data presented on Figs 2k, l and Supplementary Fig. S4b were generated by analyzing the data available

under the accession number GSE932042. Data presented on Figs 2m-p were generated by analyzing the data from GSE874112. Data presented on Fig 4e was generated by analyzing the data from GSE81538. Data presented on Figs 5a, b were generated by analyzing the data from GSE124647. Data presented on Figs 6d was generated by analyzing the data from GSE81538. Data presented on Supplementary Fig. S4c was generated by analyzing the data from GSE202203. These GEO dataset are available at GEO depository (https://www.ncbi.nlm.nih.gov/geo/).

Human research participants

Policy information about studies involving human research participants and Sex and Gender in Research.

Reporting on sex and gender	All the patients were female as mostly females are diagnosed with breast cancer. No sex- and gender-based analyses have been performed.
Population characteristics	Patients were primarily diagnosed with invasive ductal carcinoma (70%) with some invasive lobular carcinoma (11%), mixed type (9%) and others/missing (10%). Patients were underwent breast conserving surgery (21.1%), modified radical mastectomy (73.7%) or have no surgery information (5.3%). Median age at diagnosis is 37 with a range of 22-78. Patients were premenopausal (42.6%), postmenopausal (51.9%), and perimenopausal (5.6%). All patients were ER-positive and 85% were also PR-positive. The patients were diagnosed with Grade I (6.3%), Grade II (50.7%), and Grade III (43.1%). The patients were diagnosed with Stage I (17.8%), Stage II (56.8%), and Stage III (25.4%). All patients were treated with endocrine therapy with or without radiotherapy and chemotherapy.
Recruitment	Tumors from 172 ER+ breast cancer patients that were diagnosed between 2000 and 2016 at Hacettepe University School of Medicine, Ankara, Turkey were analyzed for this study. De-identified FFPE tumors were extracted from medical archives, and tissue microarrays (TMA) were prepared depending on the quality and amount of the FFPE blocks. There was no bias in patient selection.
Ethics oversight	The study was approved by the Non-Interventional Clinical Research Ethics Committee of Hacettepe University (approval no: 2020/02-40).

Note that full information on the approval of the study protocol must also be provided in the manuscript.

Field-specific reporting

Please select the one below that is the best fit for your research. If you are not sure, read the appropriate sections before making your selection.

☒ Life sciences ☐ Behavioural & social sciences ☐ Ecological, evolutionary & environmental sciences

For a reference copy of the document with all sections, see nature.com/documents/nr-reporting-summary-flat.pdf

Life sciences study design

All studies must disclose on these points even when the disclosure is negative.

Sample size	For in vitro experiments, sample sizes were chosen based on experience from previous experiments using similar techniques (Saatci et al. 2020, Nature Communications; Saatci et al, 2022, Cell Death & Differentiation; Mishra et al, 2018, Clinical Cancer Research) to reach statistically relevant results. Sample sizes for in vivo experiments were determined based on previous studies (Saatci et al, 2018, Oncogene; Saatci et al. 2020, Nature Communications; Saatci et al, 2022, Cell Death & Differentiation; Mishra et al, 2018, Clinical Cancer Research).
Data exclusions	During patient sample analysis, few of the TMA cores that have fallen off the slide during slide preparation were not stained and not included. For in vitro experiments, replicates that were identified as outliers by Grubbs method (alpha=0.1) using GraphPad software were excluded.
Replication	Experiments were repeated at least twice and reproducible results were obtained.
Randomization	In vitro samples were randomly allocated into different treatment groups. For in vivo experiments, mice were randomly allocated into different treatment groups.
Blinding	Investigators were not blinded while allocating mice into groups, during treatment, data collection or analysis because the sample names contained treatment information. No blinding was performed for the other experiments since the investigators should keep careful track of protocols, most of the experiments needed multiple treatments and the samples should first be allocated into different groups.

Reporting for specific materials, systems and methods

We require information from authors about some types of materials, experimental systems and methods used in many studies. Here, indicate whether each material, system or method listed is relevant to your study. If you are not sure if a list item applies to your research, read the appropriate section before selecting a response.

Materials & experimental systems

n/a	Involved in the study
☐	☒ Antibodies
☐	☒ Eukaryotic cell lines
☒	☐ Palaeontology and archaeology
☐	☒ Animals and other organisms
☒	☐ Clinical data
☒	☐ Dual use research of concern

Methods

n/a	Involved in the study
☒	☐ ChIP-seq
☐	☒ Flow cytometry
☒	☐ MRI-based neuroimaging

Antibodies

Antibodies used

Alexa Fluor 488 anti-mouse, Life Technologies, A-11001
Alexa Fluor 647 anti-rabbit, Life Technologies, A-31573
Beta-actin, MP Biomedicals, 691001
GAPDH, Santa Cruz, sc-47724
PDE4D, ProteinTech, 12918-1-AP
FEN1, ProteinTech, 14768-1-AP
XRCC1, Santa Cruz, sc-56254
PARP1, BD Biosciences, 556494
p-RB, Cell Signaling, 8516
HPF1, Cell Signaling, 90876
Acetyl-Histone H3 (Lys9), Cell Signaling, 9649
Histone H3, Cell Signaling, 4499
Alpha-tubulin, Santa Cruz, sc-32293
ER, Santa Cruz, sc-8002
p-H2AX (S139), Santa Cruz, sc-517348
p-Chk2 (T68), Cell Signaling, 2197
p-Chk1 (S345), Cell Signaling, 2348
p-PKA (Thr197), Cell Signaling, 4781
p-CREB (S133), Cell Signaling, 9198
p-ER (S118), Cell Signaling, 2511
p-ERK1/2 (T202/Y204), Cell Signaling, 4376
p-EGFR (Tyr845), Sigma, 07-820
p-HER2 (Y1248)-EGFR (Y1173), Cell Signaling, 2244
p-AKT (S473), Cell Signaling, 4058
AKT, Cell Signaling, 9272
Cyclin D1, Cell Signaling, 2922
Cleaved PARP, Cell Signaling, 5625
PARP, Cell Signaling, 9542
CDK4, Abcam, ab68266
ERK1/2, Cell Signaling, 4695
c-Myc, Santa Cruz, sc-40
c-Jun, Cell Signaling, 9165
ER, Active Motif, 61035
COXIV, ProteinTech, 11242-1-AP
H3S10 ADPr, BioRad, HCA357
PAR/pADPr, R&D systems, 4335-MC-100
Poly/Mono-ADP Ribose, Cell Signaling, 9649
RAD51, Abcam, ab133534
BRCA1, ProteinTech, 22362-1-AP
BRCA2, Cell Signaling, 10741

Validation

All antibodies are commercially available and validated in the literature as cited on the manufacturer's websites as well as by the datasheets they provide. Below are the validation data and application notes from suppliers' websites:
PDE4D (ProteinTech, 12 918-1-AP) antibody was validated in knockout system. FEN1 antibody (ProteinTech, 14768-1-AP) was tested in various lysates by Western blotting. XRCC1 antibody (Santa Cruz, sc-56254) was tested in humna and mouse cell lines by Western blotting. PARP1 antibody (BD Biosciences, 556494) is routinely tested by western blot analysis of untreated Jurkat T cells and Jurkat T cells induced to undergo apoptosis. p-RB antibody (Cell Signaling, 8516) was tested in human cells treated with phosphatases by Western blotting. HPF1 antibody (Cell Signaling, 90876) was tested in different human and mouse cell lines with Western blotting. Acetyl-Histone H3 (Lys9) antibody (Cell Signaling, 9649) has been validated using SimpleChIP® Enzymatic Chromatin IP Kits by the manufacturer. Histone H3 antibody (Cell Signaling, 4499) was tested by Western blot analysis of extracts from various human and mouse cell lines. Alpha-tubulin antibody (Santa Cruz, sc-32293) was tested in different human and mouse cell lines with Western blotting. ER antibody (Santa Cruz, sc-8002) was tested in different human and mouse cell lines with Western blotting. p-H2AX (S139) antibody (Santa Cruz, sc-517348) was tested in different human and mouse cell lines with Western blotting. p-Chk2 (T68) antibody (Cell Signaling, 2197) was tested by Western blot analysis of extracts from untreated or UV-treated human cells. p-Chk1 (S345) antibody (Cell Signaling, 2348) was tested by Western blot analysis of extracts from untreated or UV-treated human and mouse cells.

p-PKA (Thr197) antibody (Cell Signaling, 4781) was tested in mouse and rat cells treated with phosphatases by Western blotting. p-CREB (S133) antibody (Cell Signaling, 9198) was tested in forskolin- or FGF-treated human cells by Western blotting. p-ER (S118) antibody (Cell Signaling, 2511) was tedted by Western blotting in human cells transfected with wild-type or mutant ER alpha, stimulated with EGF and E2. p-ERK1/2 (T202/Y204) antibody (Cell Signaling, 4376) was tested in PDGF- or EGF-treated human and mouse cells by Western blotting. p-EGFR (Tyr845) antibody (Sigma, 07-820) was routinely evaluated by Western blotting and was tested in EGF-stimulated human cells by Western blotting. p-HER2 (Y1248)-EGFR (Y1173) antibody (Cell Signaling, 2244) was tested by Western blotting in extracts from human cells treated with neuregulin (NRG). p-AKT (S473) antibody (Cell Signaling, 4058) was tested in PDGF-treated mouse cells by Western blotting. AKT antibody (Cell Signaling, 9272) was knockdown-validated in human cells by Western blotting. Cyclin D1 antibody (Cell Signaling, 2922) was tested in human cells by Western blotting. Cleaved PARP antibody (Cell Signaling, 5625) was tested in human cells treated with Staurosporine by Western blotting. CDK4 antibody (Abcam, ab68266) was produced recombinantly (animal-free) for high batch-to-batch consistency and tested in human cells by Western blotting. ERK1/2 antibody (Cell Signaling, 4695) was tested in human and mouse cells by Western blotting. c-Myc antibody (Santa Cruz, sc-40) was tested in monkey cells by Western blotting. c-Jun antibody (Cell Signaling, 9165) has been validated using SimpleChIP® Enzymatic Chromatin IP Kits by the manufacturer. ER antibody (Active Motif, 61035) has been validated for use in ChIP and/or ChIP-Seq by the manufacturer. COXIV antibody (ProteinTech, 11242-1-AP) was tested by Western blotting in extracts from human and mouse cells. H3S10 ADPr antibody (BioRad, HCA357) was tested by Western blotting in human cells with PARP1 knock out and H2O2 treatment. PAR/pADPr antibody (R&D systems, 4335-MC-100) was tested in human cells treated with H2O2 by Western blotting. RAD51 antibody (Abcam, ab133534) was tested in human cells by Western blotting and immunofluorescence. BRCA1 antibody (ProteinTech, 22362-1-AP) was tested in human cells by Western blotting and was also validated in knockdown/out systems. BRCA2 antibody (Cell Signaling, 10741) was tested in human cells by Western blotting.

Eukaryotic cell lines

Policy information about cell lines and Sex and Gender in Research

Cell line source(s)	Human breast cancer cell lines, MCF-7 (HTB-22), T47D (HTB-133), ZR-75-30 (CRL-1504), MDA-MB-436 (HTB-130) normal human breast epithelial cell lines MCF-12A (CRL-3598) and MCF-10A (CRL-10317), and normal mouse fibroblast cell line, NIH3T3 (CRL-1658) were purchased from ATCC. The human breast cancer cell line MDA-MB-361 was purchased from Tissue Culture Facility (TCF) Shared Resource of MUSC. The drug resistant versions of these cell lines were derived using T47D and MCF-7 cells, culturing the parental cells in parallel. PDX primary cells were isolated from the PDX tumors of the models of interest. All the established cell lines and PDX cells are originally derived from female breast cancer patients.
Authentication	The cell lines were authenticated using STR sequencing.
Mycoplasma contamination	All cell lines were tested as negative for mycoplasma contamination using MycoAlert mycoplasma detection kit (Lonza, NJ, USA).
Commonly misidentified lines (See ICLAC register)	There are no misidentified cell lines in this study.

Animals and other research organisms

Policy information about studies involving animals; ARRIVE guidelines recommended for reporting animal research, and Sex and Gender in Research

Laboratory animals	Mice used in the study were all female. The strain used were Nu/J mice. All mice were 6-8 weeks old during tumor transplantations. All mice were maintained under a temperature-controlled environment with a 12-hour light/dark cycle and received a standard diet and water ad libitum. The animal facilities maintain centrally controlled and monitored humidity and light cycles.
Wild animals	There are no wild animals used in this study.
Reporting on sex	Since breast cancer is primarily a female disease, all mice used were female. Therefore, no sex-based analysis was performed.
Field-collected samples	There are no field-collected samples used in this study.
Ethics oversight	All the in vivo studies were carried out in accordance with the Institutional Animal Care and Use Committee of the University of South Carolina and Medical University of South Carolina.

Note that full information on the approval of the study protocol must also be provided in the manuscript.

Flow Cytometry

Plots

Confirm that:

- [x] The axis labels state the marker and fluorochrome used (e.g. CD4-FITC).
- [x] The axis scales are clearly visible. Include numbers along axes only for bottom left plot of group (a 'group' is an analysis of identical markers).
- [x] All plots are contour plots with outliers or pseudocolor plots.
- [x] A numerical value for number of cells or percentage (with statistics) is provided.

Methodology

Sample preparation	Cells were trypsinized and counted and 100,000 cells per tube with two replicates for each group were collected, centrifuged and washed once with PBS. For apoptosis assay, cell pellet was dissolved in 100 uL Annexin V binding buffer and 1.5 uL of FITC-conjugated Annexin V and DAPI were added. For cell cycle assay, cells were fixed in ethanol for 30 min on ice and stained with DAPI. Staining was done at room temperature for 15 min in the dark. Then, all samples were diluted in Annexin V binding buffer followed by analysis with flow cytometry. For the BrdU/7AAD assay, BrdU/7AAD Flow kit (BD Biosciences) was utilized based on manufacturer's instructions.
Instrument	The instruments used for data collection were the LSR II (BD) or Fortessa X-20 (BD).
Software	Data collection was done using LSR II (BD) or Fortessa X-20 (BD), and data analysis were done using FlowJo version 10.8.1, FACSDiva version 7, or De Novo Software: FCS Express version 6.
Cell population abundance	The percentage of cell populations included in the analysis were more than 90%. There were no specific impurities interfering with the analysis or acquisition.
Gating strategy	The starting cell population was selected to exclude cell debris and included more than 90% of the population. The gating for Annexin V or DAPI positivity for the apoptosis assay was determined based on unstained samples and untreated controls that are expected to be negative for Annexin V and DAPI. During cell cycle assay, the histogram of DAPI (x-axis) and cell count (y-axis) demonstrated cells at the G1, S and G2/M phases. The peak of the G1 phase of the cell cycle was gated which shows low DAPI staining compared to S and G2/M (correlating with DNA amount at G1) and highest cell count (corresponding to high percentage of cells at G1). During the Brdu/7AAD staining, each cell cycle phase, i.e. G1, S and G2/M are clearly separated based on BrdU and 7AAD co-staining as shown on the figure.

☒ Tick this box to confirm that a figure exemplifying the gating strategy is provided in the Supplementary Information.
